# Genome Analysis of an Alphabaculovirus Isolated from the Larch Looper, *Erannis ankeraria*

**DOI:** 10.3390/v14010034

**Published:** 2021-12-24

**Authors:** Long Liu, Zhilin Zhang, Chenglin Liu, Liangjian Qu, Dun Wang

**Affiliations:** 1 State Key Laboratory of Crop Stress Biology for Arid Areas, College of Plant Protection, Northwest A&F University, Yangling, Xianyang 712100, China; dragonliulong@foxmail.com; 2 Forest Protection Station, Ulanqab 012000, China; zhangzhilin1761@163.com; 3 Institute of Forest Ecology, Environment and Nature Conservation, Chinese Academy of Forestry, Beijing 100091, China; lcl@caf.ac.cn

**Keywords:** baculovirus, *Alphabaculovirus*, *Erannis ankeraria*, EranNPV, complete genome

## Abstract

The larch looper, *Erannis ankeraria* Staudinger (Lepidoptera: Geometridae), is one of the major insect pests of larch forests, widely distributed from southeastern Europe to East Asia. A naturally occurring baculovirus, Erannis ankeraria nucleopolyhedrovirus (EranNPV), was isolated from *E. ankeraria* larvae. This virus was characterized by electron microscopy and by sequencing the whole viral genome. The occlusion bodies (OBs) of EranNPV exhibited irregular polyhedral shapes containing multiple enveloped rod-shaped virions with a single nucleocapsid per virion. The EranNPV genome was 125,247 bp in length with a nucleotide distribution of 34.9% G+C. A total of 131 hypothetical open reading frames (ORFs) were identified, including the 38 baculovirus core genes and five multi-copy genes. Five homologous regions (*hrs*) were found in the EranNPV genome. Phylogeny and pairwise kimura 2-parameter analysis indicated that EranNPV was a novel group II alphabaculovirus and was most closely related to Apocheima cinerarium NPV (ApciNPV). Field trials showed that EranNPV was effective in controlling *E*. *ankeraria* in larch forests. The above results will be relevant to the functional research on EranNPV and promote the use of this virus as a biocontrol agent.

## 1. Introduction

Baculoviruses are pathogenic viruses that mainly infect insects of orders Lepidoptera, Diptera, and Hymenoptera. Baculoviruses typically infect only one or a few related insect species and are thus successfully employed as environmentally safe biopesticides for pest control [1,2,3]. Baculoviruses are also well known in biotechnology, as baculoviruses offer efficient expression systems for various applications ranging from routine expression of exogenous genes in the laboratory to vaccine production and gene therapy [4,5].

Members of the *Baculoviridae* are characterized by possessing large, circular, supercoiled, and double-stranded DNA genomes that combine with capsid proteins to form an enveloped nucleocapsid. The baculoviral genomes range from approximately 80 to 180 kbp and encode between 90 and 180 genes [6]. The production of two types of structurally and functionally distinct progeny viruses is a striking feature of the typical baculovirus life cycle. One virion phenotype is called the budded virus (BV), which is responsible for spreading infection systemically from cell to cell throughout the host, and another one is referred to as the occlusion-derived virus (ODV) transmitting infection orally from insect to insect [7]. ODVs are embedded in proteinaceous occlusion bodies (OBs), which provide the virus with physical protection in the environment. Based on the OB morphology, baculoviruses comprise nucleopolyhedroviruses (NPVs) or granuloviruses (GVs) [8]. The family *Baculoviridae* is composed of four genera: *Alphabaculovirus* (NPVs that specifically infect the insect order Lepidoptera), *Betabaculovirus* (GVs that specifically infect the insect order Lepidoptera), *Gammabaculovirus* (NPVs that specifically infect the insect order Hymenoptera), and *Deltabaculovirus* (NPVs that specifically infect the insect order Diptera) [9]. *Alphabaculovirus* can be further subdivided into two lineages, group I and group II. Group I alphabaculoviruses use GP64 as their envelope fusion protein (EFP) in the BV phenotype, whereas group II alphabaculoviruses as well as betabaculoviruses and deltabaculoviruses lack GP64 and exploit F protein as the EFP [10]. Studies on the whole genome of baculoviruses are helpful in guiding our current understanding of baculovirus, including the biotechnological use of the viruses and the revelation of intrinsic attributes critical for virus identification and classification, gene function, and insecticidal potential [11,12].

The larch looper, *Erannis ankeraria* (Lepidoptera: Geometridae), was one of the major insect pests of larch forests, widely distributed from southeastern Europe to East Asia. The larvae of this moth can lead to serious damages to trees from *Larix* spp., *Quercus* L., and *Picea* mills [13]. In the current study, Erannis ankeraria nucleopolyhedrovirus (EranNPV) was isolated from infected *E. ankeraria* larvae. EranNPV was examined by electron microscopy, and the complete genome was determined, analyzed, and compared to other fully sequenced baculovirus genomes. The size of the EranNPV genome was found to be 125,247 bp. Phylogenetic analysis showed that EranNPV was a group II alphabaculovirus and was most closely related to Apocheima cinerarium nucleopolyhedrovirus (ApciNPV). Additionally, EranNPV was effective in controlling *E*. *ankeraria* in larch forests, indicating a potential of this virus to be developed as bio-pesticide.

## 2. Materials and Methods

### 2.1. Virus Isolation

EranNPV was isolated from a larval cadaver of *E. ankeraria* collected in Ulanqab, Inner Mongolia, China, in June 2018. The virus was propagated in the third instar laboratory-reared *E. ankeraria* larvae. EranNPV OBs were purified from larval cadavers dying from infection by steps of maceration, homogenization, filtration, centrifugation, and sucrose density-gradient ultracentrifugation, as described previously [14].

### 2.2. Electron Microscopy

For the scanning electron microscope (SEM), purified OBs were observed using a scanning electron microscope (Hitachi S-3400N, Hitachi, Tokyo, Japan) at a 5 kV acceleration voltage. For the transmission electron microscope (TEM), EranNPV OBs were embedded in 2% agarose and fixed in 2.5% glutaraldehyde at 4 °C overnight. After six rinses with 0.2 mol/L phosphate buffer (pH 7.4), the samples were post-fixed in 1% osmium tetroxide at room temperature for 2 h. Fixed samples were rinsed, dehydrated, embedded, sectioned, and stained as described previously [15]. Ultrathin sections were imaged under a Hitachi HT7700 (Hitachi, Tokyo, Japan) transmission electron microscope at an accelerating voltage of 80 kV.

### 2.3. Viral DNA Isolation, Sequencing, and Assembly

Viral genomic DNA was isolated from purified OBs according to the following method. The purified OBs were resuspended in 100 mM Na_2_CO_3_ and incubated at 37 °C for 30 min. The pH value of the solution was adjusted to eight with 1 M HCl. RNase A (final concentration was 45 μg/mL) was added to the solution and incubated at 37 °C for 10 min. Virion particles were disintegrated by adding SDS (final concentration was 1%) and proteinase K (final concentration was 250 μg/mL), followed by incubation for 1 h at 37 °C. After extracting with phenol/chloroform/isoamyl alcohol (25:24:1), the viral DNA was precipitated by ethanol. The DNA concentration was determined by spectrophotometry, and the DNA purity was verified by measuring the 260/280 ratio.

EranNPV full genome sequencing was performed on both the Illumina NovaSeq 6000 system and the PromethION platform from Oxford Nanopore Technologies. For Illumina NovaSeq 6000 system, 18,064,430 clean reads were generated. The GC, Q20, and Q30 contents were 34.64%, 97.18%, and 91.97%, respectively. For the PromethION platform, a total of 33,114 reads were obtained with an N50 size of 30,295 bp, of which the average length was 30,199 bp. The Unicycler software (version 2.1.2) was used to assemble the sequence data. A high-quality genome skeleton (contig) was obtained by assembling high-accuracy Illumina reads (Q30 > 85%), followed by the connection of the contig using the Nanopore reads.

### 2.4. Genome Sequence Analysis

The open reading frames (ORFs) potentially encoding proteins with at least 50 amino acids and minimal overlap were predicted using FGENESV (http://linux1.softberry.com/berry.phtml, accessed on 18 June 2021) and the NCBI ORF Finder (http://www.ncbi.nlm.nih.gov/gorf/gorf.html, accessed on 18 June 2021). The identified ORFs were annotated according to homology by the NCBI Protein-Protein BLAST algorithm (https://blast.ncbi.nlm.nih.gov/Blast.cgi, accessed on 20 June 2021). The complete genomic data were submitted to GenBank under accession number OK091173.

Homologous regions (*hrs*) were identified using the tandem repeats finder [16] and Blast2seq [17]. The alignment of individual repeats from the *hrs* was performed using ClustalW in MEGA software (version 10.1.8) [18]. The BoxShade server (https://embnet.vital-it.ch/software/BOX_form.html, accessed on 26 June 2021) was employed to display the alignment and repeat the consensus sequence. The ViennaRNA server (http://rna.tbi.univie.ac.at/, accessed on 27 June 2021) was applied to predict the secondary structure of the conserved *hr* sequence [19].

Identities among proteins encoded by EranNPV and their homologous proteins in selected genomes were computed using BioEdit software (version 7.0.9.0) with default parameters. Gene parity plots were generated to compare ORF organization between EranNPV and the selected baculoviruses [20].

### 2.5. Phylogeny and Kimura 2-Parameter Analysis

Protein sequences encoded by the 38 core genes were extracted from 107 completely sequenced baculovirus genomes (Tables S1 and S2) and aligned using the MAFFT method with auto strategy and normal alignment mode [21]. The Gblocks were used to remove poorly aligned sites with default parameters [22]. Concatenation of alignments of 38 core protein sequences from 107 baculovirus species was implemented using PhyloSuite (version 1.2.2) [23]. The best partitioning scheme for maximum likelihood (ML) was determined by PartitionFinder2 according to the Bayesian information criterion (BIC) and a greedy search algorithm with branch lengths linked [24]. ML tree was inferred with IQ-TREE under Ultrafast bootstrap with 5000 replicates, as well as the SH-aLRT test with 1000 replicates [25,26,27]. For phylogenetic analysis of the inhibitor of apoptosis protein (IAP), the alignment of amino acid sequences and the construction of ML trees were carried out with the same methods. ModelFinder was applied to determine the best-fit model of amino acid evolution for IAP proteins with the default settings according to BIC [28]. The best-fit model for IAP was WAG+F+I+G4.

Kimura 2-parameter (K2P) pairwise distances from aligned nucleotide sequences of *polyhedrin*, *lef-8*, *lef-9*, and concatenated *polyhedrin*/*lef-8*/*lef-9*/fragments were calculated separately using the pairwise distance calculation in MEGA software (version 10.1.8). The variation among sites was set to be uniform. Gaps within the alignment were treated as pairwise deletions. K2P pairwise distances of concatenated nucleotide sequences of the 38 core genes were calculated as described previously [29].

### 2.6. Field Testing of EranNPV

As an attempt to explore the pesticidal potential of EranNPV, field trials against a natural infestation of *E. ankeraria* larvae were conducted in a planted *Larix principis-rupprechtii* forest in Ulanqab, Inner Mongolia, China, in May 2019. The concentration of EranNPV OBs was determined by a standard hemocytometer. EranNPV OBs were resuspended in tap water. The concentration of EranNPV OBs used to spray was 1.09 × 10^8^ OBs/mL. The application of the treatment was imposed on trees using a motorized sprayer (3WZ-300L, Nantong Guangyi Electromechanical Co., Ltd., Hai’an, Jiangsu Province, China) during evening hours, with the spray evenly distributed on the leaf surface until the spray droplet drops as the standard. The water spraying was set as a blank control. The plot sizes of the treatment and non-treated control were 100 m^2^. After spraying, twenty trees were selected from each plot, and one branch containing twenty larvae was selected from each tree and was covered with the insect rearing cage (35 cm long and 10 cm in diameter). The surviving larvae in the cages were recorded at 12 d post applications.

## 3. Results and Discussion

### 3.1. Ultrastructural Features of Occlusion Bodies

**Figure 1:**
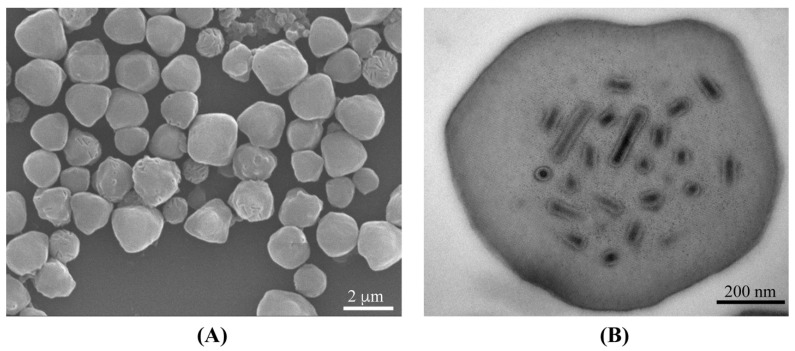
Occlusion bodies of EranNPV. (**A**) The scanning electron micrograph of occlusion bodies; (**B**) the transmission electron micrograph of ultrathin sections through occlusion bodies.

SEM revealed EranNPV OBs to be irregular polyhedral shapes (Figure 1A). The irregular shape of OBs is the typical morphological characteristic of alphabaculoviruses described in other studies, such as Mythimna unipuncta nucleopolyhedrovirus isolate KY310 (MyunNPV-KY310) [30], Cryptophlebia peltastica nucleopolyhedrovirus (CrpeNPV) [31], and Operophtera brumata nucleopolyhedrovirus (OpbuNPV) [32], etc. TEM showed that each OB contained numerous enveloped rod-shaped virions with a single nucleocapsid within the ODV (Figure 1B).

### 3.2. Features of EranNPV Genome

**Figure 2:**
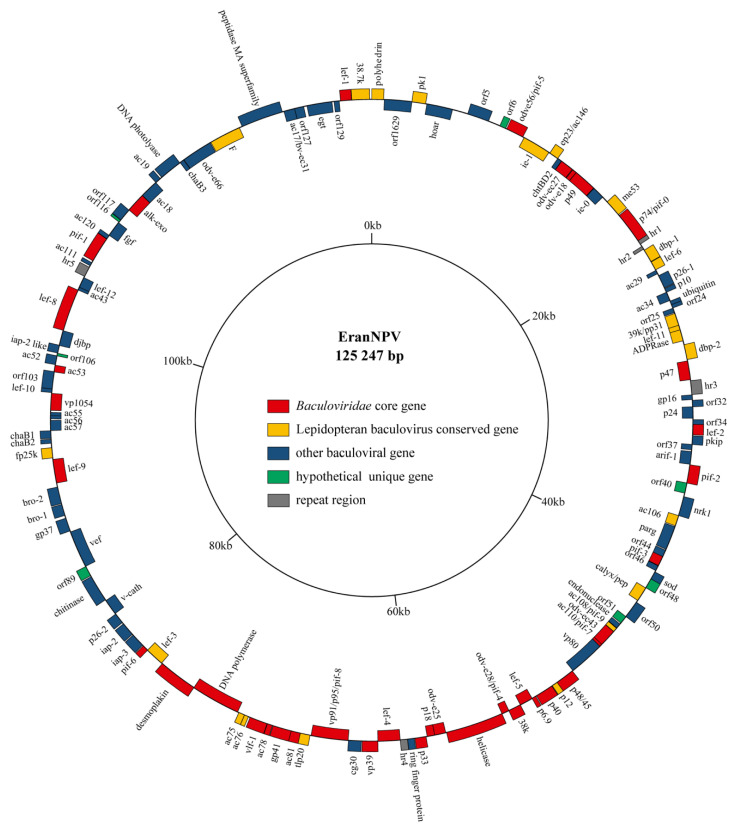
Circular map of EranNPV genome. Genes in the positive strand are drawn to the outside of the circle, and those in the negative strand are on the inside. The colors represent gene types: red represents baculovirus core genes, yellow represent genes present in all lepidopteran baculoviruses, blue represents other baculoviral genes, green represents hypothetical genes unique to EranNPV. Gray squares represent repeat regions.

The complete genome of the EranNPV isolate consisted of 125,247 bp with 34.9% GC content. The genome size and the GC content falls into the range of other sequenced baculoviruses [6]. A total of 131 putative ORFs that encode at least 50 amino acids with minimal overlaps were identified and annotated in the EranNPV genome. The coding regions covered 91.46% of the whole genome. The locations and directions of identified ORFs were illustrated in the circular genome map, and the adenine of the *polyhedrin* gene initiation codon was defined as the first nucleotide position (Figure 2). The ORFs exhibited almost equal distribution on both strands of the DNA throughout the genome, with 68 ORFs in clockwise (+) direction and 63 ORFs in anticlockwise (−) direction. EranNPV genome contained the 38 core genes that were found in all baculovirus genomes [33,34], 22 lepidopteran baculovirus conserved genes, 64 common genes that possessed homolog sequences in other baculoviruses, and 7 unique genes (*orf6*, *orf40*, *orf48*, *orf51*, *orf89*, *orf106*, and *orf116*) without homologs in *Baculoviridae* (Figure 2).

Among 131 ORFs predicted in the EranNPV genome, regions 200 bp upstream of the initiation codon were screened. Early promoters consisting of a TATA box motif followed by a 20–40 bp downstream CAKT were found in 36 ORFs. One hundred and five ORFs harbored a baculovirus late promoter element (DTAAG). Twenty-eight ORFs had both an early and late promoter motif. A total of 18 ORFs lacked any recognizable canonical promoter motif (Table S3).

### 3.3. Homologous Regions

**Figure 3:**
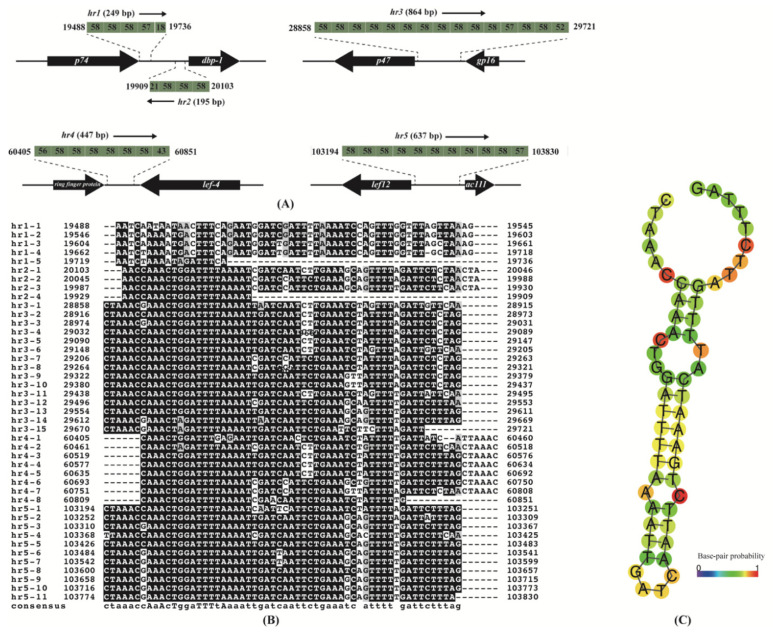
Analysis of EranNPV homologous regions (*hrs*). (**A**) The schematic diagram for location and structure of the five *hrs* in the EranNPV genome. Green blocks indicate *hrs* repeat units with the length of each unit. (**B**) Sequence alignment of EranNPV *hr* repeat units. The completely identical residues in the alignment were indicated by uppercase letters in the consensus sequence, and a majority of identical residues in the alignment were denoted using lowercase letters. (**C**) Secondary structure prediction of the conserved *hr* sequence.

Most baculovirus genomes contain repeated AT-rich sequences called homologous regions (*hrs*). *Hrs* are interspersed at different locations within the genome and characterized by comprising a number of tandem repeats that include an imperfect palindromic core [35]. The *hrs* of different baculoviruses differ widely in the sequence length, composition, and number of repeats [36]. The *hrs* play putative or demonstrated roles as origins of DNA replication and enhancers of gene transcription [37,38,39]. Five *hrs* were identified in the EranNPV genome with an AT content of 72–74%. Except for *hr2* that was positioned in the anticlockwise direction, all the *hrs* were positioned in the clockwise direction in the genome. As shown in Figure 3A, the *hrs* of the EranNPV genome were composed of 4–15 tandem repeats of about 58 bp in length. The *hr1* and *hr2* were located between *p74* and *dbp-1* and contained five and four repeat units, respectively. The *hr3* was the longest and was located at the intergenic region between *p47* and *gp16* with a total of 15 copies of tandem repeats. The *hr4* was located between *ring finger protein* and *lef-4 and* was made of eight repeat units. The *hr5* was present in the intergenic region between *lef-12* and *ac111* with 11 tandem repeats. The alignment and secondary structure prediction of the tandem repeats (Figure 3B,C) indicated that the repeat units of EranNPV *hrs* were imperfect inverted repeat sequences.

### 3.4. Phylogenetic Analysis of EranNPV

**Figure 4:**
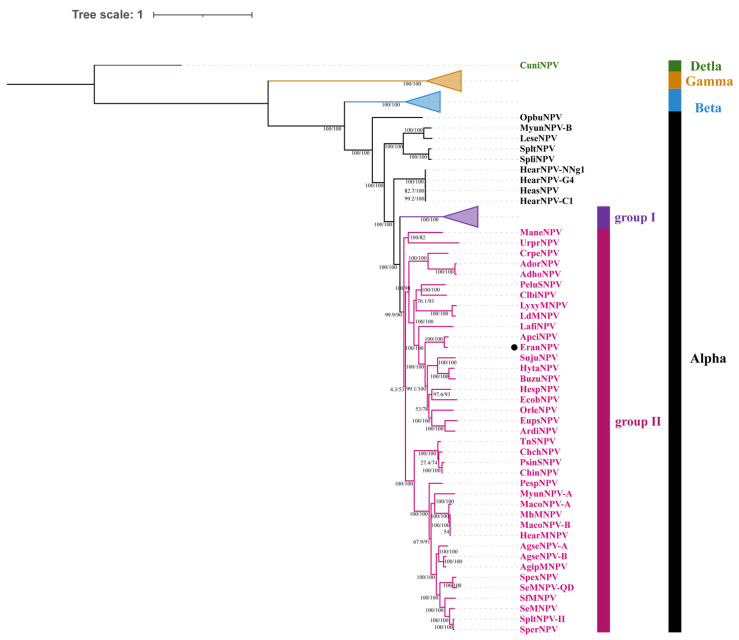
ML tree inferred from the concatenated alignments of 38 core protein sequences from 107 baculoviruses using IQ-TREE. Bootstrap support values and SH-aLRT values are indicated on branches. EranNPV is marked by the black dot.

EranNPV encoded the F protein and lacked the *gp64* gene, indicating that it was a group II alphabaculovirus. Phylogeny among EranNPV and other completely sequenced baculoviruses based on concatenated alignments of the 38 baculovirus core protein sequences confirmed that the EranNPV belonged to group II of genus *Alphabaculovirus* and shared the closest phylogenetic relationship with ApciNPV (Figure 4). From the ML phylogram, alphabaculoviruses clustered into a monophyletic group, and group I alphabaculoviruses formed a monophyletic clade within the alphabaculovirus group. However, three minor monophyletic subgroups containing nine species were placed in a basal position relative to other alphabaculoviruses. This topological structure is in accordance with previous studies [10].

Based on pairwise nucleotide distances using the K2P substitution model, two baculoviruses should be deemed as different virus species if their K2P pairwise distances of the *polyhedrin*, *lef-8*, and *lef-9* genes are larger than 0.05 substitutions/site [40]. The K2P pairwise distances of *polyhedrin*, *lef-8*, *lef-9*, and concatenated *polyhedrin*/*lef-8*/*lef-9* fragments between EranNPV and other related NPVs were more than 0.05 substitutions/site (Table 1). Additionally, pairwise K2P nucleotide distances of concatenated 38 core genes between EranNPV and other related NPVs were all larger than the threshold of 0.072 (Table 1), which was proposed by Wennmann et al. (2018) to separate two baculoviruses species. The above results indicated that EranNPV was a distinct alphabaculovirus.

### 3.5. Genome Comparison

**Figure 5:**
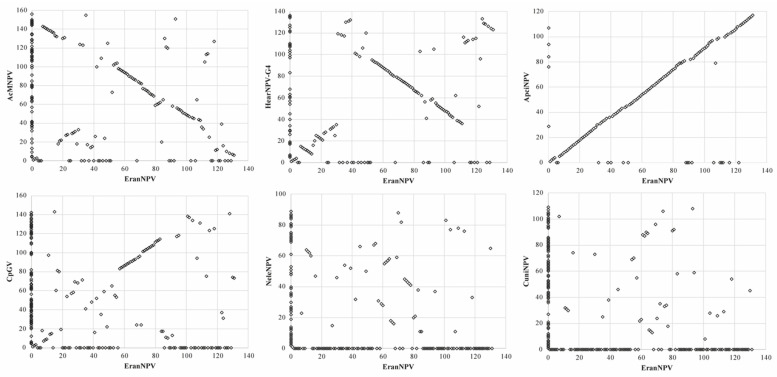
Gene parity plots analysis. Gene parity plots of EranNPV compared to AcMNPV, HearNPV-G4, ApciNPV, CpGV, NeleNPV, and CuniNPV based on ORF order. Black squares indicate the presence and relative position of ORFs in the EranNPV genome with those from other viruses.

The gene content and order of the EranNPV genome were compared with six representative baculoviruses: Autographa californica multiple nucleopolyhedrovirus (AcMNPV, group I alphabaculovirus), Helicoverpa armigera nucleopolyhedrovirus G4 (HearNPV-G4, minor group alphabaculovirus), ApciNPV (group II alphabaculovirus), Cydia pomonella granulovirus (CpGV, betabaculovirus), Neodiprion lecontei nucleopolyhedrovirus (NeleNPV, gammabaculovirus) and Culex nigripalpus nucleopolyhedrovirus (CuniNPV, deltabaculovirus). EranNPV shared 103 homologous ORFs with AcMNPV, 105 with HearNPV-G4, 114 with ApciNPV, 78 with CpGV, 47 with NeleNPV, and 39 with CuniNPV. Regarding the 38 core proteins, EranNPV shared an average amino acid identity of 43.1%, 49.9%, 82.5%, 29.4%, 20.7%, and 15.5% with the above six viruses, respectively. Gene parity plots analysis (Figure 5) revealed that the gene order of EranNPV was highly collinear with ApciNPV and partially collinear with AcMNPV, HearNPV-G4, and CpGV. However, the gene arrangement of EranNPV was noticeably divergent with NeleNPV and CuniNPV.

### 3.6. Gene Content of EranNPV

#### 3.6.1. Classification of EranNPV Genes

As in other baculoviruses, annotated ORFs of the EranNPV genome can be categorized based on their function, including genes related to replication, transcription, structure and oral infectivity, auxiliary genes, and genes with unknown function (Table 2). Fifteen genes involved in viral DNA replication were found in the EranNPV genome. Other DNA replication genes, including *helicase-2*, *DNA ligase*, *pcna*, *lef-7*, *rr1*, *rr2*, and *dUTPase*, were absent from the EranNPV genome. Except for EranNPV, some other alphabaculoviruses also lack these seven genes related to DNA replication, such as ApciNPV, Buzura suppressaria nucleopolyhedrovirus (BuzuNPV), and Adoxophyes orana nucleopolyhedrovirus (AdorNPV). Two group I alphabaculoviruses, Choristoneura occidentalis nucleopolyhedrovirus (ChocNPV) and Choristoneura rosaceana nucleopolyhedrovirus (ChroNPV), contain *pcna* but lack the remaining six genes [41]. *Helicase-2* and *DNA ligase* are considered to be implicated in DNA repair and recombination [42]. Homologs of *pcna* are only present in a few alphabaculoviruses from group I. Homologs of *lef-7* were found to be present in several alphabaculoviruses and in a few betabaculoviruses specific for noctuid hosts [43]. AcMNPV *lef-7* is a replication factor associated with the manipulation of the DNA damage response (DDR) of the host to promote efficient virus replication [44]. Homologs of *Rr1*, *rr2*, and *dUTPase* coding for enzymes related to nucleotide metabolism were found in some alphabaculoviruses and betabaculoviruses [45].

EranNPV encoded all baculovirus transcription genes except for *ie-2* and *pe38*. Additionally, 32 structural genes were present in the EranNPV genome; however, EranNPV lacked homologs to *gp50* and *gp64*.

Successful oral infection of baculoviruses relies on a multiprotein complex of *per os infectivity factors* (PIFs) on the ODV envelope [46]. The EranNPV contained all 10 baculovirus *pif* genes, *pif-0*–*pif-9*. Except for *pif-9*, all the *pif* genes encoded by EranNPV were baculoviral core genes. Besides PIFs, the EranNPV also possessed a major ODV-specific envelope protein, ODV-E66, that is conserved in all lepidopteran baculoviruses [47,48]. The ODV-E66 plays an important role in the penetration of the peritrophic membrane (PM) during oral infection [49].

Auxiliary genes are a class of genes that are not essential for viral DNA replication, gene expression, and formation of progeny virion, but they provide the virus some selective advantages in the context of species-specific virus-host interactions [50]. A total of 20 auxiliary genes were found in the EranNPV genome. Additional genes (*ptp-1*, *ptp-2*, *ctl-1*, *ctl-2*) also known as auxiliary genes were not found in EranNPV genome.

#### 3.6.2. Analysis of EranNPV Multi-Copy Genes

To date, most sequenced baculovirus genomes contain one to 16 copies of baculovirus repeated ORFs (*bro*) whose function is not well characterized [51]. Studies on the Bombyx mori nucleopolyhedrovirus (BmNPV) *bro* genes revealed that BRO proteins could bind to DNA and were related to influencing host DNA replication [52]. Two adjacent *bro* genes, *bro-1* (ORF92) and *bro-2* (ORF93), were identified in the EranNPV genome.

Homologs of DNA binding protein (*dbp*) were found in all sequenced baculovirus genomes except for CuniNPV, and multiple copies of the *dbp* gene are present in group II alphabaculoviruses. The *dbp* gene is involved in unwinding and annealing DNA during replication [53]. Two copies of the *dbp* gene were found in the EranNPV genome.

**Figure 6:**
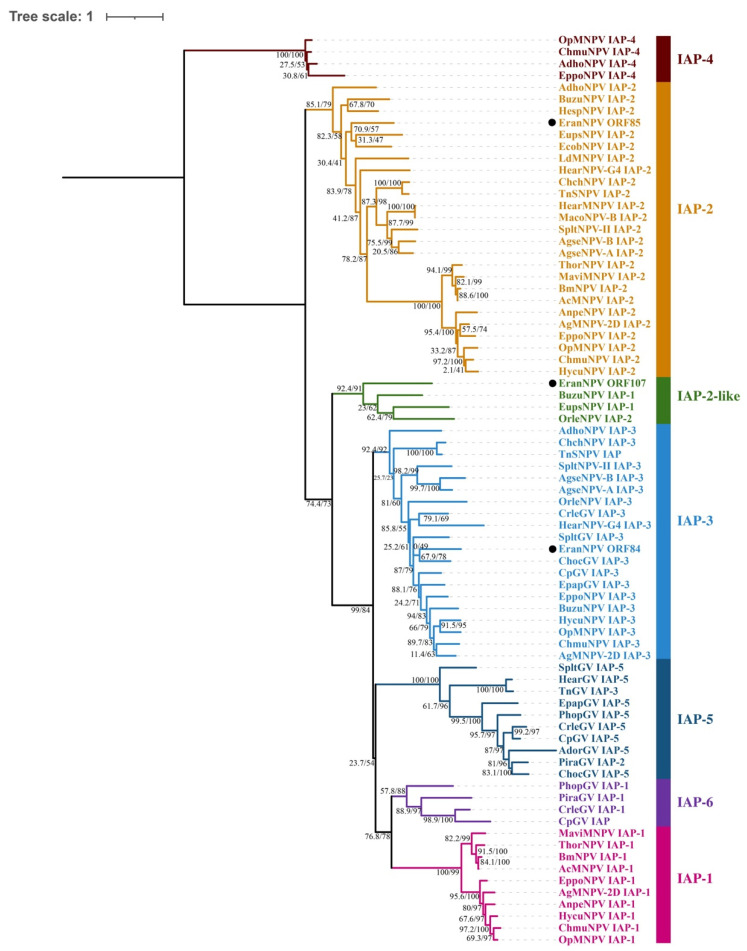
ML tree inferred from selected baculovirus IAP proteins using IQ-TREE. Bootstrap support values and SH-aLRT values are indicated on branches. IAP homologs in the EranNPV genome are marked by black dots.

To abrogate programmed cell death, baculoviruses code for two families of genes blocking apoptosis that is triggered at the early stages of infection, *p35*, and inhibitor of apoptosis (*iap*). *Iaps* are found to make up for the lack of *p35* [54,55]. Within the baculoviruses, six lineages of *iap* genes have been identified, named *iap*-*1* to *iap*-*6*. IAP-1, -2, -3, and -5 proteins each contain two well-conserved baculoviral IAP repeat (BIR) domains and a really interesting new gene (RING) domain at the C-terminal. IAP-4 proteins are composed of a ring domain and a single BIR domain that is smaller than the normal BIR domain. Betabaculoviruses IAPs containing a single BIR and RING belong to the IAP-6 lineage. A small number of alphabaculovirus IAP-2 homologs that lack a copy of BIR are referred to as IAP-2-like proteins. Various combinations of *iap-1*, *iap-2*, *iap-3*, and *iap-4* are present in alphabaculoviruses, while betabaculoviruses carry *iap-3*, *iap-5*, and *iap-6* [56]. In the EranNPV genome, three ORFs (84, 85, and 107) were found to encode IAP homologs. According to the phylogenetic analysis (Figure 6) with selected baculoviral IAPs, IAP homologs in the EranNPV genome were separated into different groups. ORF84 that had a single BIR and RING belonged to IAP-2-like groups. Both ORF85 and 107 were made up of two copies of BIR and a RING, and they were clustered into IAP-2 and IAP-3 groups, respectively.

The *p26* gene is an alphabaculovirus-specific gene with a conserved position, and some alphabaculoviruses contain a second copy of this gene. The EranNPV encoded two copies of *p26*, *p26-1*, and *p26-2*. Consistent with other group II alphabaculoviruses that have two copies of *p26*, EranNPV *p26-1* and *p26-2* were adjacent to *p10* and *iap-2*, respectively.

The baculovirus ChaB proteins are present in all completely sequenced alphabaculoviruses except Urbanus proteus nucleopolyhedrovirus (UrprNPV) and some GVs, and most alphabaculoviruses contain two copies of *ChaB*. It has been demonstrated that the homologous gene of *ChaB* encoded by HearNPV is transcribed in the early stage of infection and is involved in viral DNA replication and BV production [57]. The EranNPV was the fifth alphabaculovirus that contained three copies of *ChaB*. The other four viruses are BuzuNPV, Hemileuca sp. nucleopolyhedrovirus (HespNPV), Orgyia leucostigma nucleopolyhedrovirus (OrleNPV), and Artaxa digramma nucleopolyhedrovirus (ArdiNPV).

### 3.7. The Potential of EranNPV as Bio-Pesticide

In previously reported laboratory bioassays, the LD_50_ value of EranNPV against the third instar *E. ankeraria* larvae was 10^6^ OBs/larva (regression equation: y = 1.9621 + 0.5056x; 95% CI: 10^5.86^–10^6.16^ OBs/larva) and EranNPV was more virulent to early-instar larvae [58]. The average OB content of the second, third, fourth, and fifth instar *E. ankeraria* larvae and pupae dying from EranNPV infection were 6.96 × 10^7^, 2.76 × 10^8^, 5.34 × 10^8^, 7.46 × 10^8^ OBs/larva, and 1.77 × 10^8^ OBs/pupa, respectively. The average OB content of the fifth instar *E. ankeraria* larvae can reach up to 1.5 × 10^9^ OBs/larva [58]. Compared with EranNPV kept in the refrigerator, EranNPV remaining on the trees for one year showed a certain activity with a decrease in toxicity of 63.2%, indicating that the application of EranNPV in the forest has important implications for the prevalence of viral diseases in pest populations in the following year [58]. The LD_50_ value of EranNPV against the third instar *E. ankeraria* larvae is higher than that of some other commercial baculoviruses against their host insects, such as HearNPV-C1 [59]. However, the performance of a baculovirus in the laboratory cannot completely reflect the control efficacy in the field.

As an attempt to explore the potential of EranNPV as a bio-pesticide to reduce the use of chemical pesticides, field trials in larch forests were conducted. The adjusted mortality of *E. ankeraria* larvae was 88.8% at the concentration of 1.09 × 10^8^ OBs/mL on the twelfth day after spraying EranNPV, indicating that EranNPV was effective to control *E. ankeraria* under field conditions. However, only one concentration was used in this study; further field testing is required to determine the optimal dose of this virus for controlling *E. ankeraria* in the field. In a previous study, field trials of EranNPV against *E. ankeraria* larvae were also conducted in larch forests [60]. At a rate of 7.5 × 10^11^ OBs/ha, population decline rates were 65.9% and 90.3% at 15 and 23 d post applications, respectively. At a rate of 7.5 × 10^12^ OBs/ha, population decline rates were 68.4% and 97.8% at 15 and 23 d post applications, respectively. In the second year, four different dosages (7.5 × 10^11^, 1.5 × 10^12^, 2.25 × 10^12^, and 3 × 10^12^ OBs/ha) were used to perform field trials, and adjusted population decline rates were 72.4%, 68%, 77.5%, and 91.0% at 18 d post applications, respectively. Additionally, the control efficacy of EranNPV at the dosage of 2.25 × 10^12^/ha was similar to that of fenvalerate at the concentration of 45 mL/ha. After applying the combination of EranNPV (1.5 × 10^12^ OBs/ha) and fenvalerate (45 mL/ha), the population decline rate was 95.5% at 18 d post application [60]. Therefore, EranNPV has the potential to be developed as a microbial bio-pesticide for the management of *E. ankeraria* in the future.

## 4. Conclusions

In this work, EranNPV OBs were examined by electron microscopy, and the complete genome of this virus was sequenced and analyzed. EranNPV OBs exhibited irregular polyhedral shapes containing multiple enveloped rod-shaped virions, with a single nucleocapsid per virion. EranNPV was a novel group II alphabaculovirus and was most closely related to ApciNPV. In addition, field trials showed that EranNPV was effective in controlling *E. ankeraria*. These results provide a basis for the research on functional genomics of EranNPV as well as important data for genetic modification and developing this virus as bio-pesticide.

## Figures and Tables

**Table 1:** Pairwise distances of the nucleotide sequences of (**A**) *polyhedrin* (*polh*) and concatenated *polh*/*lef-8*/*lef-9* fragments, (**B**) *lef-9* and *lef-8*, and (**C**) concatenated nucleotide sequences of the 38 core genes among EranNPV and other related species.

**(A)**											
	***polh/lef-8/lef-9***	**1**	**2**	**3**	**4**	**5**	**6**	**7**	**8**	**9**	**10**
***polh***											
1	EranNPV	-	0.12	0.35	0.34	0.34	0.34	0.33	0.33	0.33	0.34
2	ApciNPV	0.09	-	0.36	0.35	0.34	0.35	0.34	0.33	0.35	0.34
3	SujuNPV	0.20	0.20	-	0.30	0.31	0.36	0.34	0.34	0.36	0.35
4	HytaNPV	0.22	0.21	0.23	-	0.18	0.34	0.33	0.32	0.33	0.32
5	BuzuNPV	0.21	0.20	0.24	0.17	-	0.35	0.34	0.33	0.33	0.34
6	HespNPV	0.22	0.23	0.23	0.22	0.23	-	0.34	0.34	0.35	0.35
7	EcobNPV	0.21	0.22	0.23	0.19	0.23	0.22	-	0.34	0.33	0.34
8	OrleNPV	0.21	0.20	0.22	0.19	0.21	0.21	0.21	-	0.31	0.30
9	EupsNPV	0.27	0.28	0.26	0.24	0.22	0.25	0.24	0.22	-	0.24
10	ArdiNPV	0.23	0.22	0.25	0.19	0.20	0.21	0.21	0.21	0.21	-
**(B)**											
	***lef-9***	**1**	**2**	**3**	**4**	**5**	**6**	**7**	**8**	**9**	**10**
***lef-8***											
1	EranNPV	-	0.12	0.35	0.34	0.35	0.33	0.33	0.34	0.33	0.37
2	ApciNPV	0.12	-	0.36	0.36	0.36	0.36	0.35	0.35	0.36	0.36
3	SujuNPV	0.39	0.40	-	0.31	0.32	0.37	0.32	0.34	0.38	0.35
4	HytaNPV	0.38	0.38	0.31	-	0.16	0.34	0.32	0.31	0.35	0.35
5	BuzuNPV	0.38	0.37	0.33	0.18	-	0.34	0.30	0.30	0.35	0.35
6	HespNPV	0.38	0.39	0.40	0.38	0.40	-	0.35	0.35	0.35	0.35
7	EcobNPV	0.37	0.37	0.39	0.38	0.40	0.37	-	0.33	0.33	0.36
8	OrleNPV	0.37	0.36	0.38	0.36	0.38	0.38	0.37	-	0.31	0.29
9	EupsNPV	0.35	0.36	0.38	0.34	0.36	0.37	0.37	0.34	-	0.27
10	ArdiNPV	0.36	0.36	0.38	0.35	0.37	0.39	0.37	0.33	0.24	-
**(C)**											
**38 Core Genes**		**1**	**2**	**3**	**4**	**5**	**6**	**7**	**8**	**9**	
1	EranNPV										
2	ApciNPV	0.13									
3	SujuNPV	0.49	0.48								
4	HytaNPV	0.51	0.49	0.45							
5	BuzuNPV	0.48	0.47	0.43	0.25						
6	HespNPV	0.49	0.47	0.51	0.51	0.50					
7	EcobNPV	0.49	0.47	0.50	0.51	0.50	0.50				
8	OrleNPV	0.49	0.47	0.50	0.49	0.48	0.48	0.50			
9	EupsNPV	0.49	0.48	0.51	0.50	0.50	0.49	0.50	0.46		
10	ArdiNPV	0.49	0.48	0.50	0.49	0.49	0.49	0.49	0.46	0.33	

**Table 2:** Classification of EranNPV genes.

Gene Function	Genes Present in EranNPV (ORF No.)	Genes Missing in EranNPV
Replication	*ie-1* (8), *me53* (15), *dbp-1* (17), *lef-11* (27), *dbp-2* (29), *lef-2* (35), *nrk-1* (41), *parg* (43), *endonuclease* (52), *helicase* (64), *DNA polymerase* (80), *lef-3* (82), *alk-exo* (118), *DNA photolyase* (121), *lef-1* (130)	*helicase-2, DNA ligase, dUTPase, pcna, lef7*, *rr1*, *rr2*
Transcription	*ie-0* (14), *lef-6* (18), *39k/pp31* (26), *p47* (30), *lef-5* (61), *lef-4* (69), *vlf-1* (77), *lef-9* (94), *lef-10* (102), *lef-8* (109), *lef-12* (111)	*ie-2, pe38*
Structure	*polyhedrin* (1), *orf1629* (2), *pk-1* (3), *odv-ec27* (11), *odv-e18* (12), *p49* (13), *p10* (21), *gp16* (31), *p24* (33), *pkip* (36), *calyx/pep* (49), *odv-ec43* (54), *vp80* (56), *p48/45* (57), *p12* (58), *p40* (59), *p6.9* (60), *38k* (62), *odv-e25* (65), *p18* (66), *p33* (67), *vp39* (70), *cg30* (71), *tlp-20* (73), *ac81* (74), *gp41* (75), *ac78* (76), *desmoplakin* (81), *fp25k* (95), *vp1054* (101), *ac53* (104), *F* (124), *ac17/bv-ec31* (126)	*gp50*, *gp64*
Oral infectivity	*odv-e56*/*pif-5* (7), *p74/pif-0* (16), *pif-2* (39), *pif-3* (45), *ac108/pif-9* (53), *ac110/pif-7* (55), *odv-e28/pif-4* (63), *vp91/p95/pif-8* (72), *pif-6* (83), *pif-1* (113), *odv-e66* (123)	
Auxiliary	*p26-1* (20), *ubiquitin* (23), *ADPRase* (28), *arif-1* (38), *sod* (47), *ring finger protein* (68), *iap-3* (84), *iap-2* (85), *p26-2* (86), *v-cath* (87), *chitinase* (88), *vef* (90), *gp37* (91), *bro-1* (92), *bro-2* (93), *iap-2-like* (107), *djbp* (108), *fgf* (115), *egt* (128), *38.7k* (131)	*ptp-1*, *ptp-2*, *ctl-1*, *ctl-2*
Unknown	*hoar* (4), *orf5* (5), *orf6* (6), *ep23*/*ac146* (9), *chtBD2* (10), *ac29* (19), *ac34* (22), *orf24* (24), *orf25* (25), *orf32* (32), *orf34* (34), *orf37* (37), *orf40* (40), *ac106* (42), *orf44* (44), *orf46* (46), *orf48* (48), *orf50* (50), *orf51* (51), *ac76* (78), *ac75* (79), *orf89* (89), *chaB1* (96), *chaB2* (97), *ac57* (98), *ac56* (99), *ac55* (100), *orf103* (103), *ac52* (105), *orf106* (106), *ac43* (110), *ac111* (112), *ac120* (114), *orf116* (116), *orf117* (117), *ac18* (119), *ac19* (120), *chaB3* (122), *peptidase MA superfamily* (125), *orf127* (127), *orf129* (129)	*ac145*

## Data Availability

No new data were created or analyzed in this study. Data sharing is not applicable to this article.

## Supplementary Materials

The following are available online at https://www.mdpi.com/article/10.3390/v14010034/s1, Table S1: Basic information of 106 sequenced baculovirus genomes in GenBank, Table S2: 38 core genes of 106 genomes, Table S3: EranNPV genome annotation.
