# Supplementary material for: Genome Analysis of an Alphabaculovirus Isolated from the Larch Looper, Erannis ankeraria
Source: Viruses. 2021 Dec 24;14(1):34. doi: 10.3390/v14010034 (PMC8779214; doi:10.3390/v14010034)
Supplement: Supplementary file 1 [file viruses-14-00034-s001.zip › viruses-1447187-supplementary/Table S1.pdf]

Table S1. Basic information of 106 sequenced baculovirus genomes in GenBank.

Genus	Virus [a]	Abbreviation	Accession no.	Strain/isolate	Length(bp)	ORFs	G + C (%)	Reference
Alphabaculovirus (Group I)	Autographa californica NPV	AcMNPV	NC_001623	C6	133894	156	40.7	[1]
	Anticarsia gemmatalis NPV	AgMNPV-2D	NC_008520	AgMNPV-2D	132239	158	44.5	[2]
	Anticarsia gemmatalis MNPV	AgMNPV-37	KR815466	AgMNPV-37	131855	156	44.5	[3]
	Antheraea pernyi NPV	AnpeNPV	NC_008035	Liaoning	126629	147	53.5	[4]
	Antheraea pernyi NPV	AnprNPV	LC194889	Liaoning	126593	150	53.3	-
	Antherea yammamai NPV	AnyaNPV	LC375537	Nagano	126270	152	53.3	-
	Bombyx mori NPV	BmNPV	NC_001962	T3	128413	143	40.4	[5]
	Bombyx mandarina NPV	BomaNPV	FJ882854	S1	126770	133	40.2	[6]
	Catopsilia Pomona NPV	CapoNPV	KU565883	416	128058	131	39.7	[7]
	Choristoneura fumiferana DEF MNPV	CfDEFMNPV	NC_005137	-	131160	149	45.8	[8]
	Choristoneura fumiferana MNPV	CfMNPV	NC_004778	-	129593	146	50.1	[9]
	Choristoneura murinana NPV	ChmuNPV	NC_023177	Darmstadt	124688	147	50	[10]
	Condylorrhiza vestigialis MNPV	CoveMNPV	NC_026430	-	125767	138	42.9	[11]
	Choristoneura rosaceana NPV	ChroNPV	NC_021924	NB_1	129052	149	48.6	[12]
	Cyclophragma undans NPV	CyunNPV	KT957089	Whiov	142900	147	45	[13]
	Dasychira pudibunda NPV	DapuNPV	KP747440	ML1	136761	161	54.4	[14]
	Epiphyas postvittana NPV	EppoNPV	NC_003083	-	118584	136	40.7	[15]
	Hyphantria cunea NPV	HycuNPV	NC_007767	-	132959	148	45.5	[16]
	Lonomia obliqua MNPV	LoobNPV	KP763670	SP/2000	120023	134	35.7	-
	Maruca vitrata MNPV	MaviMNPV	NC_008725	-	111953	126	38.6	[17]
	Neophasia sp. alphabaculovirus	NespNPV	MK293724	11	129018	144	44.6	-
	Orgyia pseudotsugata MNPV	OpMNPV	NC_001875	-	131995	152	55	[18]
	Oxyplax ochracea NPV	OxocNPV	NC_043529	435	113971	124	31.1	[19]

Genus	Virus [a]	Abbreviation	Accession no.	Strain/isolate	Length(bp)	ORFs	G + C (%)	Reference
	Philosamia cynthia ricini NPV	PhcyNPV	JX404026	GX-1	125376	138	53.7	[20]
	Plutella xylostella MNPV	PlxyMNPV	DQ457003	CL3	134417	153	40.7	[21]
	Rachiplusia ou MNPV	RoMNPV	AY145471	R1	131526	149	39.1	[22]
	Samia Cynthia NPV	SacyNPV	LC375538	Nagano	126094	152	53.3	-
	Samia ricini NPV	SariNPV	LC375541	Guangxi	125921	149	53.5	-
	Spilosoma obliqua NPV	SpobNPV	KY550224	IIPR	136141	135	45.4	-
	Thysanoplusia orichalcea NPV	ThorNPV	NC_019945	P2	132978	145	39.5	[23]
Alphabaculovirus (group II and other alphabaculoviruses)	Adoxophyes honmai NPV	AdhoNPV	NC_004690	ADN001	113220	125	35.6	[24]
	Adoxophyes orana NPV	AdorNPV	NC_011423	English	111724	121	35	[25]
	Agrotis ipsilon MNPV	AgipMNPV	NC_011345	Illinois	155122	163	48.6	[26]
	Agrotis segetum NPV	AgseNPV-A	NC_007921	A	147544	153	45.7	[27]
	Agrotis segetum NPV B	AgseNPV-B	NC_025960	B	148981	150	45.7	[28]
	Apocheima cinerarium NPV	ApciNPV	NC_018504	-	123876	118	45	-
	Artaxa digramma NPV	ArdiNPV	MN233792	424	161734	149	39.1	[29]
	Buzura suppressaria NPV	BuzuNPV	NC_023442	Hubei	120420	127	36.8	[30]
	Chrysodeixis chalcites NPV	ChchNPV	NC_007151	-	149622	151	39	[31]
	Chrysodeixis includens NPV	ChinNPV	KU669289	IA	140808	142	39.2	[32]
	Clanis bilineata NPV	ClbiNPV	NC_008293	DZ1	135454	129	37.7	[33]
	Cryptophlebia peltastica NPV	CrpeNPV	MH394321	SA	115728	126	37.2	[34]
	Ectropis obliqua NPV	EcobNPV	NC_008586	A1	131204	126	37.6	[35]
	Euproctis pseudoconspersa NPV	EupsNPV	NC_012639	Hangzhou	141291	139	40.3	[36]
	Helicoverpa armigera MNPV	HearMNPV	EU730893	-	154196	162	40.1	[37]
	Helicoverpa armigera NPV NNg1	HearNPV-NNg1	AP010907	NNg1	132425	143	39.1	[38]
	Helicoverpa armigera NPV	HearNPV-C1	AF303045	C1	130759	137	38.9	[39]

Genus	Virus [a]	Abbreviation	Accession no.	Strain/isolate	Length(bp)	ORFs	G + C (%)	Reference
	Helicoverpa armigera NPV G4	HearNPV-G4	NC_002654	G4	131405	135	39	[39]
	Helicoverpa assulta NPV	HeasNPV	MG569706	HasNPV-DJ00 31	129801	128	38.8	-
	Hemileuca sp. NPV	HespNPV	NC_021923	-	140633	137	38.1	[40]
	Hyposidra talaca NPV	HytaNPV	MH261376	HytaNPVIndia 001	139089	141	39.6	[41]
	Lambdina fiscellaria NPV	LafiNPV	NC_026922	GR15	157977	137	43.7	[42]
	Lymantria dispar MNPV	LdMNPV	NC_001973	-	161046	164	57.5	[43]
	Leucania separata NPV	LeseNPV	NC_008348	AH1	168041	169	48.6	[44]
	Lymantria xylina MNPV	LyxyMNPV	NC_013953	LyxyMNPV-5	156344	157	53.5	[45]
	Mamestra brassicae MNPV	MbMNPV	NC_023681	K1	152710	159	39.9	[46]
	Mamestra configurata NPV-A	MacoNPV-A	NC_003529	A	155060	169	41.7	[47]
	Mamestra configurata NPV-B	MacoNPV-B	NC_004117	B	158482	168	40	[48]
	Malacosoma Neustria NPV	ManeNPV	KY968317	ManeNPV-T2	130202	131	38.2	[49]
	Mythimna unipuncta NPV-A	MyunNPV-A	NC_043530	#7	148482	159	48.6	[50]
	Mythimna unipuncta NPV-B	MyunNPV-B	MH124167	KY310	156647	152	43.9	[51]
	Operophtera brumata NPV	OpbuNPV	MF614691	OpbuNPV-MA	119054	130	39.8	[52]
	Orgyia leucostigma NPV	OrleNPV	NC_010276	CFS-77	156179	135	39.9	[53]
	Perigonia lusca SNPV	PeluSNPV	NC_027923	-	132831	145	40	[54]
	Peridroma NPV	PespNPV	NC_024625	GR_167	151109	139	53.2	[55]
	Pseudoplusia includens SNPV	PsinSNPV	NC_026268	IE	139132	141	39.3	[56]
	Spodoptera exigua MNPV	SeMNPV-QD	MH370144	QD	128525	127	37.4	[57]
	Spodoptera exigua MNPV	SeMNPV	NC_002169	US1	135611	139	43.8	[58]
	Spodoptera frugiperda MNPV	SfMNPV	NC_009011	3AP2	131331	143	40.2	[59]
	Spodoptera eridania NPV	SperNPV	MH320559	251	149090	146	45	[60]

Genus	Virus [a]	Abbreviation	Accession no.	Strain/isolate	Length(bp)	ORFs	G + C (%)	Reference
	Spodoptera exempta NPV	SpexNPV	MH717816	244.1	129528	139	41.2	-
	Spodoptera littoralis NPV	SpliNPV	JX454574	AN1956	137998	132	44.7	[61]
	Spodoptera litura NPV	SpltNPV	NC_003102	G2	139342	141	42.8	[62]
	Spodoptera litura NPV II	SpltNPV-II	NC_011616	II	148634	147	45	-
	Sucra jujuba NPV	SujuNPV	KJ676450	473	135952	131	38.7	[63]
	Trichoplusia ni SNPV	TnSNPV	NC_007383	-	134394	145	39	[64]
	Urbanus proteus NPV	UrprNPV	NC_029997	Southern Brazil	105555	119	34.7	[65]
Betabaculovirus	Adoxophyes orana GV	AdorGV	NC_005038	-	99657	119	34.5	[66]
	Agrotis segetum GV	AgseGV	NC_039213	DA	131557	152	37	[67]
	Choristoneura occidentalis GV	ChocGV	NC_008168	-	104710	116	32.7	[68]
	Clostera anachoreta GV	ClanGV	NC_015398	ClanGV-HBHN	101487	123	44.4	[69]
	Clostera anastomosis GV	ClasGV-A	NC_022646	HENAN	101818	122	46.7	[70]
	Clostera anastomosis GV-B	ClasGV-B	KR091910	ClasGV-B	107439	123	37.8	[71]
	Cnaphalocrocis medinalis GV	CnmeGV	NC_029304	Enping	111246	118	35.2	[72]
	Cydia pomonella GV	CpGV	NC_002816	Mexican 1	123500	143	45.3	[73]
	Cryptophlebia leucotreta GV	CrleGV	NC_005068	CV3	110907	128	32.4	[74]
	Diatraea saccharalis GV	DisaGV	NC_028491	Parana-2009	98392	125	35	[75]
	Epinotia aporema GV	EpapGV	NC_018875	-	119082	132	41.5	[76]
	Erinnyis ello GV	ErelGV	NC_025257	S86	102759	130	38.7	[77]
	Helicoverpa armigera GV	HearGV	NC_010240	-	169794	179	40.8	[78]
	Hyphantria cunea GV	HycuGV	MH923363	Hc1	114825	132	39.3	[79]
	Mocis latipes GV	MolaGV	KR011718	Southern Brazil	134272	145	38.1	-
	Mythimna unipuncta GV	MyunGV	NC_033780	MyunGV#8	144673	153	49.9	[80]

Genus	Virus [a]	Abbreviation	Accession no.	Strain/isolate	Length(bp)	ORFs	G + C (%)	Reference
	Phthorimaea operculella GV	PhopGV	NC_004062	-	119217	130	35.7	[81]
	Plodia interpunctella GV	PiGV	NC_032255	Cambridge	112536	123	44.1	[82]
	Plutella xylostella GV	PlxyGV	NC_002593	K1	100999	120	40.7	[83]
	Pieris rapae GV	PiraGV	NC_013797	Wuhan	108592	120	33.2	[84]
	Pseudaletia unipuncta GV	PsunGV	NC_013772	Hawaiin	176677	183	39.8	-
	Spodoptera frugiperda GV	SpfrGV	NC_026511	VG008	140913	146	46.2	[85]
	Spodoptera litura GV	SpltGV	NC_009503	SlGV-K1	124121	136	38.8	[86]
	Trichoplusia ni GV	TnGV	KU752557	LBIV-12	175360	172	39.7	[87]
	Xestia c-nigrum GV	XecnGV	NC_002331	-	178733	181	40.7	[88]
Gamma baculovirus	Neodiprion abietis NPV	NeabNPV	NC_008252	-	84264	93	33.4	[89]
	Neodiprion lecontei NPV	NeleNPV	NC_005906	-	81755	89	33.3	[90]
	Neodiprion sertifer NPV	NeseNPV	NC_005905	-	86462	90	33.8	[91]
Delta baculovirus	Culex nigripalpus NPV	CuniNPV	NC_003084	Florida1997	108252	109	50.9	[92]

[a] EranNPV is not shown.

References

1. Ayres, M. D.; Howard, S. C.; Kuzio, J.; Lopez-Ferber, M.; Possee, R. D. The complete DNA sequence of Autographa californica nuclear polyhedrosis virus. *Virology* **1994,** *202*, 586-605.
2. de Castro Oliveira, J. V.; Wolff, J. L. C.; Garcia-Maruniak, A.; Ribeiro, B. M.; de Castro, M. E. B.; de Souza, M. L.; Moscardi, F.; Maruniak, J. E.; de Andrade Zanotto, P. M. Genome of the most widely used viral biopesticide: Anticarsia gemmatalis multiple nucleopolyhedrovirus. *J. Gen. Virol.* **2006,** *87*, 3233-3250.
3. Brito, A. F. d.; Braconi, C. T.; Weidmann, M.; Dilcher, M.; Alves, J. M. P.; Gruber, A.; Zanotto, P. M. d. A. The pangenome of the Anticarsia gemmatalis multiple nucleopolyhedrovirus (AgMNPV). *Genome Biol. Evol.* **2016,** *8*, 94-108.
4. Nie, Z. M.; Zhang, Z. F.; Dan, W.; He, P. A.; Jiang, C. Y.; Song, L.; Chen, F.; Xu, J.; Yang, L.; Yu, L. L. Complete sequence and organization of Antheraea pernyi

nucleopolyhedrovirus, a dr-rich baculovirus. *BMC Genomics* **2007,** *8*, 1-14.
5. Gomi, S.; Majima, K.; Maeda, S. Sequence analysis of the genome of Bombyx mori nucleopolyhedrovirus. *J. Gen. Virol.* **1999,** *80*, 1323-1337.
6. Xu, Y. P.; Ye, Z. P.; Niu, C. Y.; Bao, Y. Y.; Wang, W. B.; Shen, W. D.; Zhang, C. X. Comparative Analysis of the Genomes of *Bombyx mandarina* and *Bombyx mori* Nucleopolyhedroviruses. *J. Microbiol.* **2010,** *48*, 102-110.
7. Wang, J.; Zhu, Z.; Zhang, L.; Hou, D.; Wang, M.; Arif, B.; Kou, Z.; Wang, H.; Deng, F.; Hu, Z. Genome sequencing and analysis of Catopsilia pomona nucleopolyhedrovirus: a distinct species in group I *Alphabaculovirus*. *PLoS One* **2016,** *11*, e0155134.
8. Lauzon, H. A.; Jamieson, P. B.; Krell, P. J.; Arif, B. M. Gene organization and sequencing of the Choristoneura fumiferana defective nucleopolyhedrovirus genome. *J. Gen. Virol.* **2005,** *86*, 945-961.
9. De Jong, J. G.; Lauzon, H. A.; Dominy, C.; Poloumienko, A.; Carstens, E. B.; Arif, B. M.; Krell, P. J. Analysis of the Choristoneura fumiferana nucleopolyhedrovirus genome. *J. Gen. Virol.* **2005,** *86*, 929-943.
10. Rohrmann, G. F.; Erlandson, M. A.; Theilmann, D. A. Genome sequence of an alphabaculovirus isolated from *Choristoneura murinana*. *Genome Announc.* **2014,** *2*, e01135-01113.
11. Castro, M. E. B.; Ribeiro, Z. M. A.; Santos, A. C. B.; Souza, M. L.; Machado, E. B.; Sousa, N. J.; Moscardi, F. Identification of a new nucleopolyhedrovirus from naturally-infected *Condylorrhiza vestigialis* (Guenée)(Lepidoptera: Crambidae) larvae on poplar plantations in South Brazil. *J. Invertebr. Pathol.* **2009,** *102*, 149-154.
12. Thumbi, D. K.; Béliveau, C.; Cusson, M.; Lapointe, R.; Lucarotti, C. J. Comparative genome sequence analysis of *Choristoneura occidentalis* Freeman and *C. rosaceana* Harris (Lepidoptera: Tortricidae) alphabaculoviruses. *PLoS One* **2013,** *8*, e68968.
13. Zhu, Z.; Wang, J.; Wang, Q.; Yin, F.; Liu, X.; Hou, D.; Zhang, L.; Liu, H.; Li, J.; Arif, B. M. Genome characteristics of the Cyclophragma undans nucleopolyhedrovirus: a distinct species in group I of *Alphabaculovirus*. *Virol. Sin.* **2018,** *33*, 359-368.
14. Krejmer, M.; Skrzecz, I.; Wasag, B.; Szewczyk, B.; Rabalski, L. The genome of Dasychira pudibunda nucleopolyhedrovirus (DapuNPV) reveals novel genetic connection between baculoviruses infecting moths of the Lymantriidae family. *BMC Genomics* **2015,** *16*, 1-13.
15. Hyink, O.; Dellow, R. A.; Olsen, M. J.; Caradoc-Davies, K. M.; Drake, K.; Herniou, E. A.; Cory, J. S.; O'Reilly, D. R.; Ward, V. K. Whole genome analysis of the Epiphyas postvittana nucleopolyhedrovirus. *J. Gen. Virol.* **2002,** *83*, 957-971.
16. Ikeda, M.; Shikata, M.; Shirata, N.; Chaeychomsri, S.; Kobayashi, M. Gene organization and complete sequence of the Hyphantria cunea nucleopolyhedrovirus genome. *J. Gen. Virol.* **2006,** *87*, 2549-2562.
17. Chen, Y. R.; Wu, C. Y.; Lee, S. T.; Wu, Y. J.; Lo, C. F.; Tsai, M. F.; Wang, C. H. Genomic and host range studies of Maruca vitrata nucleopolyhedrovirus. *J. Gen. Virol.* **2008,** *89*, 2315-2330.
18. Ahrens, C.; Russell, R.; Funk, C.; Evans, J.; Harwood, S.; Rohrmann, G. The sequence of the Orgyia pseudotsugata multinucleocapsid nuclear polyhedrosis virus genome. *Virology* **1997,** *229*, 381-399.
19. Wang, J.; Hou, D.; Wang, Q.; Kuang, W.; Zhang, L.; Li, J.; Shen, S.; Deng, F.; Wang, H.; Hu, Z. Genome analysis of a novel group I alphabaculovirus obtained from *Oxyplax ochracea*. *PLoS One* **2018,** *13*, e0192279.
20. Qian, H.; Zhang, Y.; Wu, Y.; Sun, P.; Zhu, S.; Guo, X.; Gao, K.; Xu, A.; Wang, W. Analysis of the genomic sequence of Philosamia cynthia nucleopolyhedrin virus and comparison with Antheraea pernyi nucleopolyhedrin virus. *BMC Genomics* **2013,** *14*, 115.
21. Harrison, R. L.; Lynn, D. E. Genomic sequence analysis of a nucleopolyhedrovirus isolated from the diamondback moth, *Plutella xylostella*. *Virus Genes* **2007,** *35*, 857-873.
22. Harrison, R. L.; Bonning, B. C. Comparative analysis of the genomes of *Rachiplusia ou* and *Autographa californica* multiple nucleopolyhedroviruses. *J. Gen. Virol.* **2003,** *84*, 1827-1842.
23. Wang, Y. S.; Huang, G. H.; Cheng, X. H.; Wang, X.; Garretson, T. A.; Dai, L. Y.; Zhang, C. X.; Cheng, X. W. Genome of Thysanoplusia orichalcea multiple

nucleopolyhedrovirus lacks the superoxide dismutase gene. *J. Virol.* **2012,** *86*, 11948-11949.
24. Nakai, M.; Goto, C.; Kang, W.; Shikata, M.; Luque, T.; Kunimi, Y. Genome sequence and organization of a nucleopolyhedrovirus isolated from the smaller tea tortrix, *Adoxophyes honmai*. *Virology* **2003,** *316*, 171-183.
25. Hilton, S.; Winstanley, D. Genomic sequence and biological characterization of a nucleopolyhedrovirus isolated from the summer fruit tortrix, *Adoxophyes orana*. *J. Gen. Virol.* **2008,** *89*, 2898-2908.
26. Harrison, R. L. Genomic sequence analysis of the Illinois strain of the Agrotis ipsilon multiple nucleopolyhedrovirus. *Virus Genes* **2009,** *38*, 155-170.
27. Jakubowska, A. K.; Peters, S. A.; Ziemnicka, J.; Vlak, J. M.; van Oers, M. M. Genome sequence of an enhancin gene-rich nucleopolyhedrovirus (NPV) from *Agrotis segetum*: collinearity with Spodoptera exigua multiple NPV. *J. Gen. Virol.* **2006,** *87*, 537-551.
28. Wennmann, J. T.; Alletti, G. G.; Jehle, J. A. The genome sequence of Agrotis segetum nucleopolyhedrovirus B (AgseNPV-B) reveals a new baculovirus species within the agrotis baculovirus complex. *Virus Genes* **2015,** *50*, 260-276.
29. Li, J.; Duan, X.; Wang, Q.; Zhang, L.; Deng, F.; Wang, H.; Hu, Z.; Wang, M.; Wang, J. Genome analysis of a novel clade II. b alphabaculovirus obtained from *Artaxa digramma*. *Viruses* **2019,** *11*, 925.
30. Zhu, Z.; Yin, F.; Liu, X.; Hou, D.; Wang, J.; Zhang, L.; Arif, B.; Wang, H.; Deng, F.; Hu, Z. Genome sequence and analysis of Buzura suppressaria nucleopolyhedrovirus: a group II alphabaculovirus. *PLoS One* **2014,** *9*, e86450.
31. van Oers, M. M.; Abma-Henkens, M. H.; Herniou, E. A.; de Groot, J. C.; Peters, S.; Vlak, J. M. Genome sequence of Chrysodeixis chalcites nucleopolyhedrovirus, a baculovirus with two DNA photolyase genes. *J. Gen. Virol.* **2005,** *86*, 2069-2080.
32. Craveiro, S. R.; Santos, L. A. V.; Togawa, R. C.; Inglis, P. W.; Grynberg, P.; Ribeiro, Z. M. A.; Ribeiro, B. M.; Castro, M. E. B. Complete genome sequences of six Chrysodeixis includens nucleopolyhedrovirus isolates from Brazil and Guatemala. *Genome Announc.* **2016,** *4*, e01192-01116.
33. Zhu, S. Y.; Yi, J. P.; Shen, W. D.; Wang, L. Q.; He, H. G.; Wang, Y.; Li, B.; Wang, W. B. Genomic sequence, organization and characteristics of a new nucleopolyhedrovirus isolated from *Clanis bilineata* larva. *BMC Genomics* **2009,** *10*, 91.
34. Marsberg, T.; Jukes, M. D.; Krejmer-Rabalska, M.; Rabalski, L.; Knox, C. M.; Moore, S. D.; Hill, M. P.; Szewczyk, B. Morphological, genetic and biological characterisation of a novel alphabaculovirus isolated from *Cryptophlebia peltastica* (Lepidoptera: Tortricidae). *J. Invertebr. Pathol.* **2018,** *157*, 90-99.
35. Ma, X. C.; Shang, J. Y.; Yang, Z. N.; Bao, Y. Y.; Xiao, Q.; Zhang, C. X. Genome sequence and organization of a nucleopolyhedrovirus that infects the tea looper caterpillar, *Ectropis obliqua*. *Virology* **2007,** *360*, 235-246.
36. Tang, X.-D.; Xiao, Q.; Ma, X.-C.; Zhu, Z.-R.; Zhang, C.-X. Morphology and genome of Euproctis pseudoconspersa nucleopolyhedrovirus. *Virus Genes* **2009,** *38*, 495-506.
37. Tang, P.; Zhang, H.; Li, Y. N.; Han, B.; Wang, G. Z.; Qin, Q. L.; Zhang, Z. F. Genomic sequencing and analyses of HearMNPV-a new multinucleocapsid nucleopolyhedrovirus isolated from *Helicoverpa armigera*. *Virol. J.* **2012,** *9*.
38. Ogembo, J. G.; Caoili, B. L.; Shikata, M.; Chaeychomsri, S.; Kobayashi, M.; Ikeda, M. Comparative genomic sequence analysis of novel Helicoverpa armigera nucleopolyhedrovirus (NPV) isolated from Kenya and three other previously sequenced Helicoverpa spp. NPVs. *Virus Genes* **2009,** *39*, 261-272.
39. Zhang, C.-X.; Ma, X.-C.; Guo, Z.-J. Comparison of the complete genome sequence between C1 and G4 isolates of the Helicoverpa armigera single nucleocapsid nucleopolyhedrovirus. *Virology* **2005,** *333*, 190-199.
40. Rohrmann, G. F.; Erlandson, M. A.; Theilmann, D. A. The genome of a baculovirus isolated from *Hemileuca sp.* encodes a serpin ortholog. *Virus Genes* **2013,** *47*, 357-364.
41. Nguyen, T. T.; Suryamohan, K.; Kuriakose, B.; Janakiraman, V.; Reichelt, M.; Chaudhuri, S.; Guillory, J.; Divakaran, N.; Rabins, P.; Goel, R. Comprehensive analysis of single molecule sequencing-derived complete genome and whole transcriptome of Hyposidra talaca nuclear polyhedrosis virus. *Sci. Rep.* **2018,** *8*, 1-11.
42. Rohrmann, G. F.; Erlandson, M. A.; Theilmann, D. A. Genome sequence of an alphabaculovirus isolated from the oak looper, *Lambdina fiscellaria*, contains a putative 2-kilobase-pair transposable element encoding a transposase and a FLYWCH domain-containing protein. *Genome Announc.* **2015,** *3*, e00186-00115.

43. Kuzio, J.; Pearson, M. N.; Harwood, S. H.; Funk, C. J.; Evans, J. T.; Slavicek, J. M.; Rohrmann, G. F. Sequence and analysis of the genome of a baculovirus pathogenic for *Lymantria dispar*. *Virology* **1999,** *253*, 17-34.
44. Xiao, H.; Qi, Y. Genome sequence of Leucania seperata nucleopolyhedrovirus. *Virus Genes* **2007,** *35*, 845-856.
45. Nai, Y.-S.; Wu, C.-Y.; Wang, T.-C.; Chen, Y.-R.; Lau, W.-H.; Lo, C.-F.; Tsai, M.-F.; Wang, C.-H. Genomic sequencing and analyses of Lymantria xylina multiple nucleopolyhedrovirus. *BMC Genomics* **2010,** *11*, 116.
46. Choi, J. B.; Heo, W. I.; Shin, T. Y.; Bae, S. M.; Kim, W. J.; Kim, J. I.; Kwon, M.; Choi, J. Y.; Je, Y. H.; Jin, B. R. Complete genomic sequences and comparative analysis of Mamestra brassicae nucleopolyhedrovirus isolated in Korea. *Virus Genes* **2013,** *47*, 133-151.
47. Li, Q.; Donly, C.; Li, L.; Willis, L. G.; Theilmann, D. A.; Erlandson, M. Sequence and organization of the Mamestra configurata nucleopolyhedrovirus genome. *Virology* **2002,** *294*, 106-121.
48. Li, L.; Donly, C.; Li, Q.; Willis, L. G.; Keddie, B. A.; Erlandson, M. A.; Theilmann, D. A. Identification and genomic analysis of a second species of nucleopolyhedrovirus isolated from *Mamestra configurata*. *Virology* **2002,** *297*, 226-244.
49. Gencer, D.; Nalcacioglu, R.; Demirbag, Z.; Demir, I. Complete genome sequence analysis of the Malacosoma neustria nucleopolyhedrovirus from Turkey. *Virus Genes* **2018,** *54*, 706-718.
50. Harrison, R. L.; Mowery, J. D.; Rowley, D. L.; Bauchan, G. R.; Theilmann, D. A.; Rohrmann, G. F.; Erlandson, M. A. The complete genome sequence of a third distinct baculovirus isolated from the true armyworm, *Mythimna unipuncta*, contains two copies of the *lef-7* gene. *Virus Genes* **2018,** *54*, 297-310.
51. Harrison, R. L.; Mowery, J. D.; Bauchan, G. R.; Theilmann, D. A.; Erlandson, M. A. The complete genome sequence of a second alphabaculovirus from the true armyworm, *Mythimna unipuncta*: implications for baculovirus phylogeny and host specificity. *Virus Genes* **2019,** *55*, 104-116.
52. Harrison, R. L.; Rowley, D. L.; Mowery, J. D.; Bauchan, G. R.; Burand, J. P. The Operophtera brumata nucleopolyhedrovirus (OpbuNPV) represents an early, divergent lineage within genus *Alphabaculovirus*. *Viruses* **2017,** *9*, 307.
53. Thumbi, D. K.; Eveleigh, R. J.; Lucarotti, C. J.; Lapointe, R.; Graham, R. I.; Pavlik, L.; Lauzon, H. A.; Arif, B. M. Complete sequence, analysis and organization of the Orgyia leucostigma nucleopolyhedrovirus genome. *Viruses* **2011,** *3*, 2301-2327.
54. Ardisson-Araújo, D. M.; Lima, R. N.; Melo, F. L.; Clem, R. J.; Huang, N.; Báo, S. N.; Sosa-Gómez, D. R.; Ribeiro, B. M. Genome sequence of Perigonia lusca single nucleopolyhedrovirus: insights into the evolution of a nucleotide metabolism enzyme in the family Baculoviridae. *Sci. Rep.* **2016,** *6*, 24612.
55. Rohrmann, G. F.; Erlandson, M. A.; Theilmann, D. A. A distinct group II alphabaculovirus isolated from a *Peridroma* species. *Genome Announc.* **2015,** *3*, e00185-00115.
56. Craveiro, S. R.; Inglis, P. W.; Togawa, R. C.; Grynberg, P.; Melo, F. L.; Ribeiro, Z. M. A.; Ribeiro, B. M.; Báo, S. N.; Castro, M. E. B. The genome sequence of Pseudoplusia includens single nucleopolyhedrovirus and an analysis of *p26* gene evolution in the baculoviruses. *BMC Genomics* **2015,** *16*, 127.
57. Chen, Y. J.; Qi, B. X.; Zheng, G. L.; Zhang, Y.; Deng, F.; Wan, F. H.; Li, C. Y. Identification and genomic sequence analysis of a new Spodoptera exigua multiple nucleopolyhedrovirus, SeMNPV-QD, isolated from Qingdao, China. *J. Invertebr. Pathol.* **2019,** *160*, 8-17.
58. IJkel, W. F.; Van Strien, E. A.; Heldens, J. G.; Broer, R.; Zuidema, D.; Goldbach, R. W.; Vlak, J. M. Sequence and organization of the Spodoptera exigua multicapsid nucleopolyhedrovirus genome. *J. Gen. Virol.* **1999,** *80*, 3289-3304.
59. Harrison, R. L.; Puttler, B.; Popham, H. J. Genomic sequence analysis of a fast-killing isolate of Spodoptera frugiperda multiple nucleopolyhedrovirus. *J. Gen. Virol.* **2008,** *89*, 775-790.
60. Harrison, R. L.; Rowley, D. L. Complete genome sequence of an alphabaculovirus from the southern armyworm, *Spodoptera eridania*. *Microbiol. Resour. Ann.* **2019,** *8*, e01277-18.
61. Breitenbach, J. E.; El-Sheikh, E.-S. A.; Harrison, R. L.; Rowley, D. L.; Sparks, M. E.; Gundersen-Rindal, D. E.; Popham, H. J. Determination and analysis of the genome sequence of Spodoptera littoralis multiple nucleopolyhedrovirus. *Virus Res.* **2013,** *171*, 194-208.

62. Pang, Y.; Yu, J.; Wang, L.; Hu, X.; Bao, W.; Li, G.; Chen, C.; Han, H.; Hu, S.; Yang, H. Sequence analysis of the Spodoptera litura multicapsid nucleopolyhedrovirus genome. *Virology* **2001,** *287*, 391-404.
63. Liu, X.; Yin, F.; Zhu, Z.; Hou, D.; Wang, J.; Zhang, L.; Wang, M.; Wang, H.; Hu, Z.; Deng, F. Genomic sequencing and analysis of Sucra jujuba nucleopolyhedrovirus. *PLoS One* **2014,** *9*, e110023.
64. Willis, L. G.; Siepp, R.; Stewart, T. M.; Erlandson, M. A.; Theilmann, D. A. Sequence analysis of the complete genome of Trichoplusia ni single nucleopolyhedrovirus and the identification of a baculoviral photolyase gene. *Virology* **2005,** *338*, 209-226.
65. Santos, E. R.; Oliveira, L. B.; Peterson, L.; Sosa-Gómez, D. R.; Ribeiro, B. M.; Ardisson-Araújo, D. M. The complete genome sequence of the first hesperiid-infecting alphabaculovirus isolated from the leguminous pest *Urbanus proteus* (Lepidoptera: Hesperiidae). *Virus Res.* **2018,** *249*, 76-84.
66. Wormleaton, S.; Kuzio, J.; Winstanley, D. The complete sequence of the Adoxophyes orana granulovirus genome. *Virology* **2003,** *311*, 350-365.
67. Alletti, G. G.; Eigenbrod, M.; Carstens, E. B.; Kleespies, R. G.; Jehle, J. A. The genome sequence of Agrotis segetum granulovirus, isolate AgseGV-DA, reveals a new Betabaculovirus species of a slow killing granulovirus. *J. Invertebr. Pathol.* **2017,** *146*, 58-68.
68. Escasa, S. R.; Lauzon, H. A.; Mathur, A. C.; Krell, P. J.; Arif, B. M. Sequence analysis of the Choristoneura occidentalis granulovirus genome. *J. Gen. Virol.* **2006,** *87*, 1917-1933.
69. Liang, Z.; Zhang, X.; Yin, X.; Cao, S.; Xu, F. Genomic sequencing and analysis of Clostera anachoreta granulovirus. *Arch. Virol.* **2011,** *156*, 1185-1198.
70. Liang, Z.; Zhang, X.; Yin, X.; Song, X.; Shao, X.; Wang, L. Comparative analysis of the genomes of Clostera anastomosis (L.) granulovirus and Clostera anachoreta granulovirus. *Arch. Virol.* **2013,** *158*, 2109-2114.
71. Yin, F.; Zhu, Z.; Liu, X.; Hou, D.; Wang, J.; Zhang, L.; Wang, M.; Kou, Z.; Wang, H.; Deng, F. The complete genome of a new betabaculovirus from *Clostera anastomosis*. *PLoS One* **2015,** *10*, e0132792.
72. Zhang, S.; Zhu, Z.; Sun, S. F.; Chen, Q. J.; Deng, F.; Yang, K. Genome sequencing and analysis of a granulovirus isolated from the Asiatic rice leafroller, Cnaphalocrocis medinalis. *Virol. Sin.* **2015,** *30*, 417-424.
73. Luque, T.; Finch, R.; Crook, N.; O'Reilly, D. R.; Winstanley, D. The complete sequence of the Cydia pomonella granulovirus genome. *J. Gen. Virol.* **2001,** *82*, 2531-2547.
74. Lange, M.; Jehle, J. A. The genome of the Cryptophlebia leucotreta granulovirus. *Virology* **2003,** *317*, 220-236.
75. Ardisson-Araújo, D. M.; Melo, F. L.; Clem, R. J.; Wolff, J. L.; Ribeiro, B. M. A betabaculovirus-encoded *gp64* homolog codes for a functional envelope fusion protein. *J. Virol.* **2016,** *90*, 1668-1672.
76. Ferrelli, M. L.; Salvador, R.; Biedma, M. E.; Berretta, M. F.; Haase, S.; Sciocco-Cap, A.; Ghiringhelli, P. D.; Romanowski, V. Genome of Epinotia aporema granulovirus (EpapGV), a polyorganotropic fast killing betabaculovirus with a novel thymidylate kinase gene. *BMC Genomics* **2012,** *13*, 548.
77. Ardisson-Araújo, D. M. P.; de Melo, F. L.; de Souza Andrade, M.; Sihler, W.; Báo, S. N.; Ribeiro, B. M.; de Souza, M. L. Genome sequence of Erinnyis ello granulovirus (ErelGV), a natural cassava hornworm pesticide and the first sequenced sphingid-infecting betabaculovirus. *BMC Genomics* **2014,** *15*, 856.
78. Harrison, R. L.; Popham, H. J. Genomic sequence analysis of a granulovirus isolated from the Old World bollworm, *Helicoverpa armigera*. *Virus Genes* **2008,** *36*, 565-581.
79. Gencer, D.; Bayramoglu, Z.; Nalcacioglu, R.; Demirbag, Z.; Demir, I. Genome sequence analysis and organization of the Hyphantria cunea granulovirus (HycuGV-Hc1) from Turkey. *Genomics* **2020,** *112*, 459-466.
80. Harrison, R. L.; Rowley, D. L.; Mowery, J.; Bauchan, G. R.; Theilmann, D. A.; Rohrmann, G. F.; Erlandson, M. A. The complete genome sequence of a second distinct betabaculovirus from the true armyworm, *Mythimna unipuncta*. *PLoS One* **2017,** *12*, e0170510.
81. Taha, A.; Nour-el-Din, A.; Croizier, L.; Lo, M.; Croizier, G. Comparative analysis of the granulin regions of the Phthorimaea operculella and Spodoptera littoralis granuloviruses. *Virus Genes* **2000,** *21*, 147-155.
82. Harrison, R. L.; Rowley, D. L.; Funk, C. J. The complete genome sequence of Plodia interpunctella granulovirus: evidence for horizontal gene transfer and discovery of an unusual inhibitor-of-apoptosis gene. *PLoS One* **2016,** *11*, e0160389.

83. Hashimoto, Y.; Hayakawa, T.; Ueno, Y.; Fujita, T.; Sano, Y.; Matsumoto, T. Sequence analysis of the Plutella xylostella granulovirus genome. *Virology* **2000,** *275*, 358-372.
84. Zhang, B.-Q.; Cheng, R.-L.; Wang, X.-F.; Zhang, C.-X. The genome of Pieris rapae granulovirus. *J. Virol.* **2012,** *86*, 9544.
85. Cuartas, P. E.; Barrera, G. P.; Belaich, M. N.; Barreto, E.; Ghiringhelli, P. D.; Villamizar, L. F. The complete sequence of the first Spodoptera frugiperda betabaculovirus genome: a natural multiple recombinant virus. *Viruses* **2015,** *7*, 394-421.
86. Wang, Y.; Choi, J. Y.; Roh, J. Y.; Liu, Q.; Tao, X. Y.; Park, J. B.; Kim, J. S.; Je, Y. H. Genomic sequence analysis of granulovirus isolated from the tobacco cutworm, *Spodoptera litura*. *PLoS One* **2011,** *6*, e28163.
87. de los Ángeles Bivian-Hernández, M.; López-Tlacomulco, J.; Mares-Mares, E.; Ibarra, J. E.; Del Rincón-Castro, M. C. Genomic analysis of a *Trichoplusia ni* betabaculovirus (TnGV) with three different viral enhancing factors and two unique genes. *Arch. Virol.* **2017,** *162*, 3705-3715.
88. Hayakawa, T.; Ko, R.; Okano, K.; Seong, S.-I.; Goto, C.; Maeda, S. Sequence analysis of the Xestia c-nigrum granulovirus genome. *Virology* **1999,** *262*, 277-297.
89. Duffy, S. P.; Young, A. M.; Morin, B.; Lucarotti, C. J.; Koop, B. F.; Levin, D. B. Sequence analysis and organization of the Neodiprion abietis nucleopolyhedrovirus genome. *J. Virol.* **2006,** *80*, 6952-6963.
90. Lauzon, H. A.; Lucarotti, C. J.; Krell, P. J.; Feng, Q.; Retnakaran, A.; Arif, B. M. Sequence and organization of the Neodiprion lecontei nucleopolyhedrovirus genome. *J. Virol.* **2004,** *78*, 7023-7035.
91. Garcia-Maruniak, A.; Maruniak, J. E.; Zanotto, P. M.; Doumbouya, A. E.; Liu, J.-C.; Merritt, T. M.; Lanoie, J. S. Sequence analysis of the genome of the Neodiprion sertifer nucleopolyhedrovirus. *J. Virol.* **2004,** *78*, 7036-7051.
92. Afonso, C.; Tulman, E.; Lu, Z.; Balinsky, C.; Moser, B.; Becnel, J.; Rock, D.; Kutish, G. Genome sequence of a baculovirus pathogenic for Culex nigripalpus. *J. Virol.* **2001,** *75*, 11157-11165.
