# Supplementary material for: Genome Analysis of an Alphabaculovirus Isolated from the Larch Looper, Erannis ankeraria
Source: Viruses. 2021 Dec 24;14(1):34. doi: 10.3390/v14010034 (PMC8779214; doi:10.3390/v14010034)
Supplement: Supplementary file 1 [file viruses-14-00034-s001.zip › viruses-1447187-supplementary/Table S3.pdf]

Table S3. EranNPV genome annotation.

ORF no.	Name [a]	Start	Str. [b]	End	Length(aa)	Prom. Motif [c]	ORF no. (amino acid identity, %)					
							AcMNPV	HearNPV-G4	CpGV	CuniNPV	NeleNPV	ApciNPV
1	*polyhedrin*	1	+	741	246	E, L	8 (88.6)	1 (90.2)	1 (54.8)		1 (47.3)	1 (99.1)
2	*orf1629*	785	-	1710	569	L	9 (14.0)	2 (14.4)	2 (5.2)			118 (39.7)
3	*pk-1*	2502	+	3359	285	E, L	10 (38.1)	3 (39.3)	3 (28.3)			117 (88.7)
4	*hoar*	3443	-	5068	541	E		4 (15.3)				116 (46.4)
5	*orf5*	6047	+	7411	454	E, L						
6	*orf6*	8178	+	8567	129	L						
7	***odv-e56/pif-5***	8646	+	9752	368	L	148 (55.5)	15 (51.0)	18 (44.5)	102 (21.2)	23 (31.0)	115 (83.9)
8	*ie-1*	9854	-	11644	596	E	147 (23.4)	14 (33.6)	7 (9.9)			114 (63.4)
9	*ep23/ac146*	11712	+	12305	197	L	146 (32.6)	13 (32.0)	8 (21.9)			113 (63.4)
10	*chtBD2*	12312	-	12590	92	L	145 (34.7)	12 (60.8)	9 (31.3)		64 (22.0)	112 (91.3)
11	***odv-ec27***	12607	-	13437	276	L	144 (43.1)	11 (51.0)	97 (21.5)	32 (14.8)	63 (16.4)	111 (71.3)
12	***odv-e18***	13475	-	13744	89	L	143 (35.9)	10 (61.7)	14 (36.2)	31 (12.9)	62 (18.9)	110 (96.6)
13	***p49***	13753	-	15198	481	L	142 (45.0)	9 (56.5)	15 (29.0)	30 (10.3)	60 (20.6)	109 (96.2)
14	*ie-0*	15226	-	15996	256	E, L	141 (23.9)	8 (31.9)				108 (92.5)
15	*me53*	16394	+	17455	353	L	139 (16.8)	16-17 (21.5)	143 (15.9)			107 (81.1)
16	***p74/pif-0***	17534	+	19489	651	L	138 (56.4)	20 (52.7)	60 (37.1)	74 (31.4)	47 (2.3)	106 (86.9)
	hr1	19488	+	19736								
	hr2	19909	-	20103								
17	*dbp-1*	20188	+	21006	272	None	25 (17.7)	25 (25.1)	81 (10.7)			105 (82.3)
18	*lef6*	21031	+	21594	187	L	28 (19.3)	24 (28.2)	80 (8.9)			104 (58.0)
19	*ac29*	21632	-	21862	76	E, L	29 (31.5)	23 (38.1)	19 (28.2)			103 (89.4)
20	*p26-1*	22008	+	22832	274	L	136 (29.2)	22 (42.7)				102 (89.0)
21	*p10*	22873	+	23139	88	E, L	137 (21.2)	21 (51.1)				101 (89.7)

ORF no.	Name [a]	Start	Str. [b]	End	Length(aa)	Prom. Motif [c]	ORF no. (amino acid identity, %)					
							AcMNPV	HearNPV-G4	CpGV	CuniNPV	NeleNPV	ApciNPV
22	*ac34*	23141	-	23695	184	L	34 (20.8)	27 (39.9)				100 (87.6)
23	*ubiquitin*	23741	+	24004	87	L	35 (67.8)	28 (68.1)	54 (65.9)			99 (71.4)
24	*orf24*	23970	+	24191	73	L						98 (86.3)
25	*orf25*	24208	-	24495	95	None						97 (69.0)
26	*39k/pp31*	24557	-	25357	266	E, L	36 (30.5)	31 (34.1)	57 (13.4)			96 (87.7)
27	*lef-11*	25329	-	25700	234	L	37 (26.2)	32 (40.1)	58 (30.1)		15 (16.8)	95 (94.3)
28	*ADPRase*	25628	-	26332	234	E, L	38 (46.6)	33 (46.9)	69 (36.3)			94 (95.7)
29	*dbp-2*	26584	+	27522	312	L	25 (23.5)	25 (29.6)				93 (89.2)
30	***p47***	27610	-	28788	392	None	40 (51.6)	35 (43.5)	68 (35.9)	73 (15.0)	46 (22.3)	92 (95.1)
	hr3	28858	+	29721								
31	*gp16*	29739	-	30029	96	L	130 (32.0)	119 (29.7)				90 (95.8)
32	*orf32*	30080	+	30478	132	E, L						
33	*p24*	30481	-	31188	235	L	129 (35.1)	118 (47.0)	71 (18.9)			89 (88.3)
34	*orf34*	31281	-	31637	118	L						88 (82.7)
35	***lef-2***	31576	+	32220	214	L	6 (35.1)	117 (35.7)	41 (15.6)	25 (15.4)	54 (16.5)	87 (80.0)
36	*pkip*	32288	+	32824	178	E, L	24 (14.5)	130 (27.0)				86 (85.6)
37	*orf37*	32826	-	33155	109	E						85(84.4)
38	*arif-1*	33265	-	34050	261	L	21 (13.7)	131 (19.4)				
39	***pif-2***	34086	+	35234	382	L	22 (60.0)	132 (66.6)	48 (49.2)	38 (44.5)	52 (44.0)	84 (77.4)
40	*orf40*	35231	-	35872	213	None						
41	*nrk-1*	36101	+	37312	403	None	33 (13.7)		16 (14.9)			83 (74.6)
42	*ac106*	37322	-	37978	218	L	106 (14.2)	101 (48.4)	52 (16.8)		32 (17.5)	82 (93.1)
43	*parg*	38046	-	39548	500	L		100 (20.8)				81(62.6)
44	*orf44*	39616	-	40056	146	L						80 (82.8)

ORF no.	Name [a]	Start	Str. [b]	End	Length(aa)	Prom. Motif [c]	ORF no. (amino acid identity, %)					
							AcMNPV	HearNPV-G4	CpGV	CuniNPV	NeleNPV	ApciNPV
45	***pif-3***	40049	-	40669	206	L	115 (43.5)	98 (47.8)	35 (28.1)	46 (25.9)	66 (26.7)	79 (90.2)
46	*orf46*	40696	-	41022	108	E, L						78 (66.9)
47	*sod*	41117	+	41641	174	L	31 (58.0)	106 (59.7)	59 (43.1)			77 (85.7)
48	*orf48*	41619	+	42242	207	L						
49	*calyx/pep*	42268	-	43191	307	L	131 (14.7)	120 (34.0)	22 (12.0)		50 (8.1)	76 (37.4)
50	*orf50*	43325	+	44425	366	L						75 (89.0)
51	*orf51*	44433	-	45334	169	L						
52	*endonuclease*	45023	-	45334	103	L	79 (30.5)		65 (22.3)			74 (81.5)
53	*ac108/pif-9*	45361	-	45627	88	L	108 (29.5)	95 (37.2)				73 (81.5)
54	***odv-ec43***	45639	-	46715	358	E, L	109 (46.3)	94 (58.5)	55 (28.1)	69 (12.2)	67 (19.6)	72 (95.5)
55	***ac110/pif-7***	46715	-	46882	55	E, L	110 (29.8)	93 (50.0)	53 (29.0)	70 (16.6)	68 (13.0)	71 (90.9)
56	*vp80*	46884	-	49133	749	L	104 (16.8)	92 (12.2)				70 (69.6)
57	***p48/45***	49185	+	50366	393	L	103 (39.3)	91 (49.3)	83 (30.4)	55 (9.9)	31 (14.3)	69 (94.1)
58	*p12*	50359	+	50727	122	L	102 (23.0)	90 (27.3)	84 (11.0)			68 (90.9)
59	***p40***	50766	+	51893	375	L	101 (35.4)	89 (41.3)	85 (19.5)	22 (9.5)	29 (14.8)	67 (91.2)
60	***p6.9***	51950	+	52210	86	L	100 (37.9)	88 (54.1)	86 (20.9)	23 (18.4)	28 (22.0)	66 (93.0)
61	***lef-5***	52204	-	53037	277	E	99 (43.8)	87 (45.0)	87 (37.6)	88 (10.2)	55 (24.3)	65 (84.8)
62	***38k***	53044	+	53841	265	L	98 (36.8)	86 (48.2)	88 (30.9)	87 (6.9)	56 (25.2)	64 (91.3)
63	***odv-e28/pif-4***	53834	-	54301	155	L	96 (52.6)	85 (54.9)	89 (29.0)	90 (18.6)	57 (22.4)	63 (83.1)
64	***helicase***	54309	+	57938	1209	L	95 (41.4)	84 (47.3)	90 (22.2)	89 (11.5)	58 (18.6)	62 (51.4)
65	***odv-e25***	57980	-	58690	233	L	94 (40.0)	82 (52.5)	91 (39.5)	15 (11.7)	18 (14.3)	61 (76.8)
66	***p18***	58692	-	59165	157	L	93 (45.1)	81 (57.0)	92 (27.4)	13 (8.6)	17 (16.3)	60 (87.8)
67	***p33***	59239	+	59928	229	L	92 (44.0)	80 (52.7)	93 (30.9)	14 (14.7)	16 (22.4)	59 (96.0)
68	*ring finger protein*	59975	+	60391	138	L			24 ()			58 (50.3)

ORF no.	Name [a]	Start	Str. [b]	End	Length(aa)	Prom. Motif [c]	ORF no. (amino acid identity, %)					
							AcMNPV	HearNPV-G4	CpGV	CuniNPV	NeleNPV	ApciNPV
	hr4	60405	+	60851								
69	***lef-4***	60854	-	62200	462	None	90 (42.7)	79 (46.8)	95 (28.6)	96 (16.5)	59 (21.3)	57 (88.8)
70	***vp39***	62241	+	63212	323	L	89 (37.1)	78 (42.2)	96 (22.1)	24 (11.7)	88 (18.1)	56 (73.2)
71	*cg30*	63270	+	64064	264	E	88 (19.6)	77 (20.7)	24 (10.3)			55 (67.2)
72	***vp91/p95/pif-8***	64090	-	66414	774	E, L	83 (37.0)	76 (40.8)	101 (12.0)	35 (18.9)	82 (21.1)	54 (82.4)
73	*tlp-20*	66473	+	67141	222	E	82 23.1)	75 (39.9)	102 (12.0)			53 (83.4)
74	***ac81***	67089	+	67691	200	None	81 (44.6)	74 (47.5)	103 (39.6)	106 (12.6)	45 (28.5)	52 (68.1)
75	***gp41***	67678	+	68847	389	L	80 (41.5)	73 (44.8)	104 (23.8)	33 (11.2)	44 (16.8)	51 (90.6)
76	***ac78***	68887	+	69219	110	L	78 (35.8)	72 (43.8)	105 (15.9)	34 (9.9)	43 (15.4)	50 (85.4)
77	***vlf-1***	69243	+	70364	373	L	77 (69.2)	71 (66.3)	106 (30.6)	18 (19.5)	42 (23.5)	49 (61.1)
78	*ac76*	70476	+	70736	86	E, L	76 (39.0)	70 (72.0)	107 (33.7)		41 (22.9)	48 (97.6)
79	*ac75*	70744	+	71121	125	L	75 (24.0)	69 (25.0)	108 (16.0)			47 (87.2)
80	***DNA polymerase***	71211	-	74318	1035	E, L	65 (39.4)	67 (52.2)	111 (30.3)	91 (14.9)	20 (23.2)	46 (50.4)
81	***desmoplakin***	74320	+	76779	819	E, L	66 (14.1)	66 (17.0)	112 (9.0)	92 (4.5)	21 (9.9)	45 (19.8)
82	*lef-3*	76839	-	78020	393	None	67 (26.9)	65 (29.3)	113 (11.8)			43 (81.7)
83	***pif-6***	78022	+	78396	124	L	68 (22.3)	64 (51.1)	114 (21.7)	58 (16.6)	38 (19.5)	42 (86.2)
84	*iap-3*	78454	+	79314	286	L	27 (24.5)	103 (28.2)	17 (41.3)		11 (14.6)	41 (13.7)
85	*iap-2*	79387	+	80241	284	L	71 (24.4)	62 (33.3)	17 (15.2)		11 (12.0)	41 (68.1)
86	*p26-2*	80344	+	81015	223	E	136 (20.0)					40 (86.0)
87	*v-cath*	81061	-	82047	328	L	127 (63.1)	56 (44.1)	11 (40.8)			39 (46.3)
88	*chitinase*	82108	+	83793	561	E, L	126 (69.6)	41 (65.2)	10 (10.1)			
89	*orf89*	83866	+	84507	213	E, L						
90	*vef*	84523	-	86856	777	L						
91	*gp37*	86928	+	88647	275	L	64 (46.4)	58 (56.7)	13 (36.4)			37 (89.4)

ORF no.	Name [a]	Start	Str. [b]	End	Length(aa)	Prom. Motif [c]	ORF no. (amino acid identity, %)					
							AcMNPV	HearNPV-G4	CpGV	CuniNPV	NeleNPV	ApciNPV
92	*bro-1*	87946	+	88647	233	None		59 (21.7)				
93	*bro-2*	88712	+	89761	349	L	2 (49.1)	105 (13.7)		108 (8.6)		36 (15.1)
94	***lef-9***	90014	-	91501	495	L	62 (63.8)	55 (69.3)	117 (52.9)	59 (15.9)	37 (31.8)	34 (93.9)
95	*fp25k*	91600	+	92220	206	L	61 (50.9)	53 (56.6)	118 (22.1)			33 (95.6)
96	*chaB2*	92500	+	92751	83	E, L	60 (38.2)	52 (34.8)				32 (83.7)
97	*chaB1*	92810	+	93274	154	L	59 (21.4)	51 (26.7)				31 (89.8)
98	*ac57*	93290	-	93922	210	L	57 (29.3)	50 (33.1)				30 (69.5)
99	*ac56*	94016	-	94267	83	L	56 (13.8)	49 (31.3)				29 (59.4)
100	*ac55*	94209	-	94415	68	None	55(30.7)	48 (51.4)				28 (80.5)
101	***vp1054***	94544	-	95602	352	E, L	54 (38.9)	47 (46.5)	138 (24.4)	8 (16.0)	83 (16.8)	27 (87.8)
102	*lef-10*	95653	+	95880	75	L	53a (11.2)	46 (11.6)	137 (10.9)			26 (17.0)
103	*orf103*	95883	+	96956	357	L		44 (22.2)				24 (71.3)
104	***ac53***	96945	-	97364	139	E, L	53 (45.7)	43 (43.8)	134 (17.7)	28 (3.8)	77 (11.2)	23 (87.0)
105	*ac52*	97402	+	97956	184	L	52 (13.6)	42 (27.9)				22 (75.3)
106	*orf106*	97953	-	98120	55	E, L						
107	*iap-2 like*	98138	+	98629	163	E, L	71 (18.5)	62 (16.1)	94 (17.3)		11 (5.7)	41 (14.1)
108	*djbp*	98618	-	99562	314	E, L	51 (11.3)	39 (13.1)				21 (54.7)
109	***lef-8***	99589	+	102246	885	None	50 (62.8)	38 (69.4)	131 (48.4)	26 (19.3)	78 (29.5)	20 (93.4)
110	*ac43*	102264	-	102452	62	L	43 (31.1)	37 (31.2)				
111	*lef-12*	102449	-	103111	220	None	41 (22.5)	36 (34.6)				
	hr5	103194	+	103830								
112	*ac111*	103942	+	104157	71	None	111 (70.4)	116 (32.8)				
113	***pif-1***	104296	+	105897	533	L	119 (52.3)	111 (45.1)	75 (32.3)	29 (23.4)	76 (25.9)	19 (86.3)
114	*ac120*	105915	+	106163	82	L	120 (22.7)	112 (28.7)				18 (70.3)

ORF no.	Name [a]	Start	Str. [b]	End	Length(aa)	Prom. Motif [c]	ORF no. (amino acid identity, %)					
							AcMNPV	HearNPV-G4	CpGV	CuniNPV	NeleNPV	ApciNPV
115	*fgf*	106175	-	107152	325	E, L	32 (12.5)	113 (17.2)	123 (8.8)			17 (74.1)
116	*orf116*	107196	+	107354	52	None						
117	*orf117*	107372	+	108157	261	E, L						16 (83.2)
118	***alk-exo***	108176	-	109390	404	None	133 (38.5)	114 (44.5)	125 (30.5)	54 (17.3)	33 (23.2)	15 (82.7)
119	*ac18*	109404	-	110549	381	None	18 (19.6)					14 (80.9)
120	*ac19*	110561	+	110926	121	L	19 (19.8)	115 (26.1)				13 (90.0)
121	*DNA photolyase*	111042	+	112475	477	L						11 (59.0)
122	*chaB3*	112508	-	112780	90	L		52 (34.4)				
123	*odv-e66*	112796	-	114733	645	L	46 (24.5)	96 (41.1)	37 (25.7)			10 (82.5)
124	*F*	114787	-	116781	664	E, L	23 (14.8)	133 (5.0)	31 (22.2)			9 (94.7)
125	*peptidase MA superfamily*	116973	+	119624	883	L		129 (25.3)				8 (28.8)
126	*ac17/bv-ec31*	119730	-	120410	226	None	17 (15.3)	128 (24.0)				7 (76.9)
127	*orf127*	120414	-	120989	191	None						6 (76.1)
128	*egt*	121199	-	122764	521	E	15 (45.2)	126 (51.3)	141 (33.5)			5 (90.9)
129	*orf129*	122902	-	123219	105	L						4 (89.5)
130	***lef-1***	123308	+	123991	227	L	14 (33.6)	124 (46.9)	74 (35.5)	45 (15.9)	65 (23.4)	3 (84.2)
131	*38.7k*	124006	+	125112	368	L	13 (24.6)	123 (29.7)	73 (8.3)			2 (88.1)

[a] ORFs are listed by their common name or the AcMNPV ortholog and core genes are shown in Bold.

[b] ORF direction is represented by + (clockwise) or – (anticlockwise).

[c] The presence of the following conserved promoter motifs is indicated: E, early promoter motif (TATA box followed by CAKT motif 20-40 bp downstream) within 200 bp of the initiation codon; L, late promoter motif DTAAG within 200 bp of the initiation codon.
